# Separation of the effects of two reduced height (*Rht*) genes and genomic background to select for less Fusarium head blight of short-strawed winter wheat (*Triticum aestivum* L.) varieties

**DOI:** 10.1007/s00122-022-04219-4

**Published:** 2022-09-24

**Authors:** Félicien Akohoue, Silvia Koch, Jörg Plieske, Thomas Miedaner

**Affiliations:** 1 State Plant Breeding Institute, University of Hohenheim, Stuttgart, Germany; 2 SGS INSTITUT FRESENIUS GmbH, TraitGenetics Section, Am Schwabeplan 1b, 06466 Seeland OT Gatersleben, Germany

## Abstract

**Key message:**

FHB resistance shared pleiotropic loci with plant height and anther retention. Genomic prediction allows to select for genomic background reducing FHB susceptibility in the presence of the dwarfing allele *Rht-D1b*.

**Abstract:**

With the high interest for semi-dwarf cultivars in wheat, finding locally adapted resistance sources against Fusarium head blight (FHB) and FHB-neutral reduced height (*Rht*) genes is of utmost relevance. In this study, 401 genotypes of European origin without/with dwarfing alleles of *Rht-D1* and/or *Rht24* were analysed across five environments on FHB severity and the morphological traits such as plant height (PH), anther retention (AR), number of spikelets per ear, ear length and ear density. Data were analysed by combined correlation and path analyses, association mapping and coupling single- and multi-trait genome-wide association studies (ST-GWAS and MT-GWAS, respectively) and genomic prediction (GP). All FHB data were corrected for flowering date or heading stage. High genotypic correlation (*r*_*g*_ = 0.74) and direct path effect (0.57) were detected between FHB severity and anther retention (AR). Moderate correlation (*r*_*g*_ = − 0.55) was found between FHB severity and plant height (PH) with a high indirect path via AR (− 0.31). Indirect selection for FHB resistance should concentrate on AR and PH. ST-GWAS identified 25 quantitative trait loci (QTL) for FHB severity, PH and AR, while MT-GWAS detected six QTL across chromosomes 2A, 4D, 5A, 6B and 7B conveying pleiotropic effects on the traits. *Rht-D1b* was associated with high AR and FHB susceptibility. Our study identified a promising positively acting pleiotropic QTL on chromosome 7B which can be utilized to improve FHB resistance while reducing PH and AR. *Rht-D1b* genotypes having a high resistance genomic background exhibited lower FHB severity and AR. The use of GP for estimating the genomic background was more effective than selection of GWAS-detected markers. We demonstrated that GP has a great potential and should be exploited by selecting for semi-dwarf winter wheat genotypes with higher FHB resistance due to their genomic background.

**Supplementary Information:**

The online version contains supplementary material available at 10.1007/s00122-022-04219-4.

## Introduction

Wheat (*Triticum aestivum* L.) is one of the most cultivated cereal crops worldwide and serves as a staple food for millions of people. However, the production of wheat is hampered by several diseases, including Fusarium head blight (FHB) caused by numerous *Fusarium* species, with *Fusarium graminearum* Schwabe and *F. culmorum* (W.G. Smith) Sacc. being the most predominant in Central Europe. FHB causes severe yield losses and contaminates the grains with several mycotoxins including deoxynivalenol (DON) which is one of the most frequently detected in wheat (Righetti et al. 2021; Topi et al. 2021). DON makes the grain unsuitable for flour and malt and is also toxic for non-ruminant animals (Windels 2000). Damages due to FHB in wheat are likely to increase with the rising temperatures and higher atmospheric carbon dioxide (CO_2_) caused by climate change (Timmusk et al. 2020; Miedaner and Juroszek 2021; Hay et al. 2022). Fungicides and DON-reducing technologies used as traditional measures to control FHB disease increase production costs, with no significant positive return on grain yield (Wilson et al. 2018). Most effective and environmentally friendly strategies integrate appropriate agronomic practices and enhanced host resistance.

Breeding for resistance against FHB in European winter wheat faces several challenges related to the complex genetic architecture of the trait (Jiang et al. 2015; Ruan et al. 2020) and requires intensive breeding effort and accurate breeding strategies (Buerstmayr et al. 2020). FHB resistance is controlled by multiple medium- and small-effect quantitative trait loci (QTL) which are vulnerable to the changing environmental conditions due to QTL-environment interactions (Miedaner and Juroszek 2021). To date, more than 500 QTL have been reported for FHB from different breeding populations and were further clustered into 65 QTL with unique physical positions through a recent meta-analysis by Venske et al. (2019). Additionally, morphological traits such as plant height and anther retention or extrusion were shown to passively contribute to FHB resistance (Mesterházy 1995; Buerstmayr et al. 2020; Xu et al. 2020), and relationships between FHB resistance and morphological traits have been extensively reported using correlation coefficients (Buerstmayr and Buerstmayr 2015; Steiner et al. 2019b; Ruan et al. 2020; Xu et al. 2020). Although correlation analysis helps measure the degree of association between two traits, it does not explain causes and effects of the relationship (Dewey and Lu 1959; Ozukum et al. 2019). In addition, the existence of strong correlation between two traits might be due to the presence of one or many other traits which strengthen the complexity of the interactions. Understanding the complex interactions between FHB resistance and morphological traits requires the use of appropriate statistical approaches like path analysis, also referred to as structural equation modelling (SEM) or covariance structural equation modelling (CSEM).

Path analysis is a causal multivariate modelling approach which complements simple correlation analysis by unravelling the nature and magnitude of the observed relationships among traits (Wright 1934). It exploits observed correlations to estimate standardized direct and indirect effects contrary to the standard multivariate modelling which ignores causal relationships among variables and combines all effects together (Valente et al. 2013). Path effects estimated using correlations are unitless and interpreted as standardized regression slopes, allowing for comparison of the relative importance of different variables (Stage et al. 2010). Therefore, a direct path effect of a particular morphological trait on FHB resistance would indicate how much an increase of one unit in the standard deviation of that trait directly changes the standard deviation of FHB resistance. On the contrary, an indirect path effect would indicate how much a particular morphological trait changes the standard deviation of FHB resistance depending on the presence of other morphological traits. The selection for FHB resistance using the indirect path of a trait requires the simultaneous integration of one or several other traits. Based on comparison between the two types of effect, the breeder can decide which paths and morphological traits are more important to be considered for improving FHB resistance using correlated traits for higher genetic progress. Hence, a combined correlation and path analysis has several advantages including setting reliable criteria for multiple-trait selection, minimizing risks of components compensation and guiding in planning of experiments (Usman et al. 2017; Khan et al. 2022). Path analysis was used by researchers to depict complex associations among yield and related traits in several crop species including wheat (Baye et al. 2020; Hinson et al. 2022) and maize (Toebe and Cargnelutti 2013).

Plant height in wheat is controlled by at least 150 QTL scattered across the whole wheat genome (Mao et al. 2010), and 25 *Rht* genes with more than 30 dwarfing alleles are reported (Sanchez-Garcia and Bentley 2019). They became very popular after their introduction during 1960s and 1970s to initiate the “Green Revolution” in developing countries (Hedden 2003) but also to breed short-strawed wheat in industrial countries. Major *Rht* genes are *Rht-B1* (syn. *Rht1*), *Rht-D1* (syn. *Rht2*) and *Rht24*, located on chromosomes 4B, 4D and 6A, respectively (Würschum et al. 2017; Tian et al. 2022). The wild type is named “a” and the height-reducing mutant “b”. *Rht-D1b* and *Rht24b* were more frequently found in Central European wheat germplasm than *Rht-B1b*. *Rht* genes have been widely used in commercial wheat breeding to develop semi-dwarf varieties which are preferred to tall genotypes because of their lodging resistance, higher nitrogen fertilizer use efficiency, increased tillers, higher harvest index and grain yield (Zhao et al. 2022).

Unfortunately, *Rht-B1b* and *Rht-D1b* were associated with FHB susceptibility and lower anther extrusion (Lu et al. 2013; Hales et al. 2020). Miedaner et al. (2019) and Zhang et al. (2021) suggested the use of major FHB-resistant QTL through marker-assisted introgression to counterbalance the negative FHB effect of semi-dwarf genotypes. Meanwhile, it has also been demonstrated that major non-adapted FHB resistance QTL (*Fhb1*, *Fhb2*, *Fhb5*) provide only a partial resistance (Su et al. 2019), and their introgression was not equally effective across different backgrounds (Brar et al. 2019) and requires considerable breeding efforts. Breeders must also consider a larger number of medium- and small-effect QTL in locally adapted cultivars for all traits analysed here, so-called genomic background (Brar et al. 2019) in order to aim for local, short-strawed wheat cultivars with higher FHB resistance. The genomic background refers to all locally adapted genes which are likely to influence the phenotype controlled by a particular gene of interest (Yoshiki and Moriwaki 2006). In wheat, all genes of a genotype which are capable of influencing the reduction effect of major *Rht* genes on plant height can be considered as genomic background for the plant height in that genotype and similarly for FHB resistance and AR. Numerous medium- and small-effect loci have been reported for FHB resistance and anther retention, but their potential to counterbalance the negative effect major *Rht* genes such as *Rht-D1* and possible exploitation in practical breeding have been poorly investigated.

Herter et al. (2018) evaluated segregating materials derived from a biparental cross between two German winter wheat cultivars, “Solitär x Bussard”, and demonstrated that *Rht24b* was not associated with FHB resistance. This was recently confirmed in a large European winter wheat germplasm by Miedaner et al. (2022) who also found that *Rht24b* did not affect FHB resistance. Therefore, strategies to develop FHB-resistant varieties with semi-dwarfing alleles should reconsider the choice of such *Rht* genes. This gives the opportunity to develop marker arrays and strategies to facilitate the exploitation of FHB-neutral *Rht* genes in breeding programmes. However, given the complex interactions among morphological traits and their passive contribution to FHB resistance (Buerstmayr et al. 2020), a clear understanding of the effect of *Rht24b* on morphological traits, like anther retention or extrusion, is required. Furthermore, the choice of *Rht* genes could also be driven by breeding objectives and the extent to which FHB-neutral *Rht* genes reduces the plant height compared with other genes.

In this study, we aimed to (i) describe the nature and magnitude of the complex interactions between FHB resistance and morphological traits, including plant height, anther retention, number of spikelets per ear, ear length and ear density using combined correlation and path analyses; (ii) dissect the genetic architecture of FHB severity, plant height and anther retention, thus uncovering the genetic basis of the complex interactions among these traits through a joint implementation of single- and multi-trait genome-wide association study (GWAS), and genomic prediction (GP); and (iii) separate for each trait, the effects of *Rht* genes and genomic background (GB), evaluating the potential of GB in selecting FHB-resistant genotypes with short *Rht* alleles based on the single-trait GWAS and genetic estimated breeding values (GEBV) from the GP. GB refers to all QTL affecting the traits, except those associated with plant height on chromosomes 4D and 6A corresponding to *Rht-D1* and *Rht24* genes, respectively.

## Materials and methods

### Plant materials and field experiments

The materials consisted of 401 winter wheat cultivars from European origin (Table S1). These cultivars were evaluated in Germany at three locations in 2020 and two in 2021, resulting in five environments (location × year combinations) in total. In 2020, the cultivars were evaluated in Hohenheim (HOH) near Stuttgart (9.12° E, 48.42° N; 400 m above sea level [a.s.l]), Oberer Lindenhof (OLI) near Reutlingen (9.18° E, 48.28° N; 700 m a.s.l), and Wohlde (WOH) near Bergen (9.98° E, 52.80° N; 80 m a.s.l). In 2021, field experiments were conducted in HOH and OLI only. At each location, the cultivars were randomized using an incomplete lattice design with two replicates. Experimental units were planted in double rows of 0.9 m length in WOH and single rows of 1.2 m length in HOH and OLI. Experiments were sown with a density of 40 kernels/row in WOH and 60 kernels/row in HOH and OLI.

### Artificial inoculations

Inoculum of the highly aggressive single-spore isolate FC46 (IPO 39-01) of *F. culmorum* (Snijders and Perkowski 1990) was produced on autoclaved wheat kernels as described in detail by Miedaner et al. (1995, 1996). Prior to inoculation, the Fusarium suspension was diluted to a concentration of 2 × 10^5^ spores mL^−1^. Approximately 100 mL m^−2^ of the diluted inoculum was applied using an adapted agricultural sprayer (Hege 75, Waldenbuch, Germany). Inoculations were repeated four to five times at intervals of two to three days to inoculate each genotype at least once during mid-anthesis. The first inoculations were done when early cultivars started flowering.

### Phenotypic data collection

Eight traits were recorded: Fusarium head blight severity (FHB severity, %), plant height (PH, cm), anther retention (AR, %), ear length (EL, cm), number of spikelets per ear (NS, spikelets ear^−1^), ear density (ED, spikelets cm^−1^), days to flowering (DTF, days) and heading stage (HS). Days to flowering, the number of days when 75% of heads showed extruded anthers after May 1, was assessed in all environments except in WOH. Heading stage (1–9) was recorded on one a single day (different dates in different environments) and is equal to BBCH stage 51–59 (Meier 2001). Plant height was recorded for each plot after flowering. FHB severity was rated as the percentage of infected spikelets (0–100%) of each head, averaged across all plants of a plot. The first rating started at the onset of symptom development about 15–20 days after inoculation and was repeated in intervals of three to five days until the first signs of ripening. We rated each time the total number of infected spikelets per plot. This rating approach at different stages in the development of the plants under field conditions helped to evaluate the combination of both type I (incidence) and type II (symptom development) FHB resistance in one number (Nannuru et al. 2022). Anther retention was assessed as percentage of retained anthers per spike according to Atashi-Rang and Lucken (1978). Five spikes per plot were harvested five to seven days after flowering and frozen in paper bags to do the assessment in off-season. Retained anthers of two lateral florets of eight central spikelets were counted on each of the five spikes. In contrast to Atashi-Rang and Lucken (1978), anthers located between the lemma and palea, and partially extruded, were counted as retained. From the same spikes, the number of spikelets per ear (NS_*i*_) was counted and ear (spike) length (EL_*i*_, cm) was measured. Ear density ED_*i*_ was calculated as the number of spikelets per centimetre:

1 $${\text{ED}}_{i} { = }\frac{{{\text{NS}}_{i} }}{{{\text{EL}}_{i} }}$$

AR, EL and ED were estimated plot-wise by averaging values over the five spikes.

### Genotyping and molecular analysis

DNA isolation and genotyping were done by SGS Institut Fresenius, TraitGenetics Section (https://traitgenetics.com/index.php/contact, Gatersleben, Germany) from seed samples of the 401 genotypes. Genotyping was done using an Illumina 25 K Infinium single-nucleotide polymorphism (SNP) array which produced a total of 24,145 SNP markers. About 58 and 14% of the markers overlapped, respectively, with 90 K iSelect and 820 K Axiom^®^ arrays, which are publicly available at CerealsDB (http://www.cerealsdb.uk.net/cerealgenomics/CerealsDB) (Wilkinson et al. 2020). SNPs were filtered by removing markers with minor allele frequency (MAF) < 0.05 and call rate < 80%. This narrowed down the marker data to 19,969 polymorphic and high-quality SNPs with a total of 0.7% of missing data. SNPs were coded as − 1, 0 and 1 corresponding to AA, Aa and aa. A represented the major allele, while a denoted the minor allele. Heterozygous loci were replaced with missing values, and the data were imputed using the Wright’s equilibrium approach (Wright 1922). SNPs filtering, coding and imputation were conducted using the *raw.data* function in the snpReady R package (Granato et al. 2018). Start and end physical positions (Table S6) of the markers sequences were obtained by blasting against the International Wheat Genome Sequencing Consortium (IWGSC) reference sequence (IWGSC RefSeq) v.2.1 which is accessible at the Research Unit in Genomics-Info (URGI) of the French National Institute for Agriculture, Food and Environment (INRAE) (https://wheat-urgi.versailles.inra.fr/Seq-Repository/Assemblies) (Zhu et al. 2021).

### Phenotypic data analysis

For each plot, arithmetic mean of repeated ratings of FHB severity was calculated and included in the statistical analysis as described by Mesterházy (1995). Estimated individual value was validated when the corresponding coefficient of variation of error was below 5%. Outliers were removed from the data using the Bonferroni–Holm method (Bernal-Vasquez et al. 2016). Variance components, genetic coefficient of variation, broad-sense heritability and best linear unbiased estimations (BLUEs) were calculated for each of the eight traits, based on a mixed linear model:

2 $$y_{ijkl} \, = \,g_{i} \, + \,e_{j} \, + \,b_{k} \, + \, r_{l} \, + \, ge_{ij} \, + \, \varepsilon_{ijkl}$$

where *y*_*ijkl*_ is the phenotype of the *i*th genotype in the *j*th environment within the *k*th block of the *l*th replicate; *g*_*i*_ is the effect of the *i*th genotype; *e*_*j*_ is the effect of the *j*th environment; *ge*_*ij*_ is the effect of the interaction between the genotype and the *j*th environment; *b*_*k*_ is the effect of the *k*th block; *r*_*l*_ is the effect of the *l*th replicate; and *ε*_*ijkl*_ is the residual error on the phenotype of the *i*th genotype in the *j*th environment within the *k*th block of the *l*th replicate. To adjust for the impact of repeated artificial inoculations on early flowering genotypes (higher inoculum dose after flowering), days to flowering was added as a cofactor (fixed effect) to the mixed linear model for calculating FHB severity. Because days to flowering was not assessed in WOH, the model was further extended by adding heading stage as a second cofactor in order to correct FHB severity. This correction reduced the correlation coefficient between FHB severity and days to flowering from − 0.32 to − 0.16. Similarly, the correlation between FHB severity and heading stage was reduced from 0.29 to 0.11. The genotype was treated as fixed effect, while block, replicate, environment and genotype-environment interaction were included as random effects. Variance components were firstly estimated for all genotypes as one group. Secondly, genotypes were grouped based on *Rht* genes and variances components were estimated to describe the extend of genetic variation within each group for PH, FHB severity and AR. Prior to genotypes grouping, a single-trait genome-wide association study was conducted on each trait to identify significant markers associated with PH on chromosomes 4D and 6A, which correspond to *Rht-D1* and *Rht24* genes. Based on the presence/absence of *Rht-D1b* and *Rht24b*, genotypes were categorized into four *Rht* groups, namely NoRht, *Rht24b*, *Rht-D1b* and *Rht24b* + *Rht-D1b*. NoRht was composed of genotypes with tall alleles of both genes. *Rht24b* included genotypes carrying *Rht24b* only, while *Rht-D1b* was constituted of genotypes with *Rht-D1b* only. Similarly, *Rht24b* + *Rht-D1b* included genotypes possessing semi-dwarfing alleles of both genes. Dummy variables (0, 1) as described by Piepho et al. (2006) were created to separate genotypes, and genotype-dummy interaction was added to the model to estimate variance components within each *Rht* group. For dummy = 1, the interaction between genotype and dummy estimates variance components within the respective group. Significance of variance components was calculated using a likelihood ratio test (LRT) based on factor-wise comparisons of a model with all factors and a model without the respective factor (Stram and Lee 1994; Morrell 1998). Environment-specific (heterogeneous) variances were fitted for replicate, block and residual effects. Harmonic means of environment-specific residual variances were calculated and reported. The mixed linear models were fitted using the ASReml-R package v.4.1.0 (Gilmour et al. 2015). Only adjusted means (BLUEs) of the corrected FHB severity and other traits across the five environments (location × year combinations) were considered for further analyses (Table S1).

Broad-sense heritability (*H*^*2*^) was estimated for each trait following the procedure described by Piepho and Möhring (2007):

3 $$H^{2} = \frac{{\sigma_{G}^{2} }}{{\sigma_{G}^{2} + \frac{{\overline{v}_{\Delta \ldots }^{{{\text{BLUE}}}} }}{2}}}$$

where $\sigma_{G}^{2}$ is the genotypic variance and $\bar{v}_{{\Delta \ldots }}^{{{\text{BLUE}}}}$ is the mean variance of the difference of two adjusted means (BLUEs). The genetic coefficient of variation (CV_*G*_) was calculated for each trait as follows:

4 $${\text{CV}}_{G} = \frac{{\sqrt {\sigma_{G}^{2} } }}{{\overline{x}}}$$

where $\sigma_{G}^{2}$ is the genotypic variance and ${\overline{\text{x}}}$ is the mean of BLUEs of the trait. Coefficient of variation of error (CV_*ε*_) was also calculated for each trait by replacing the genotypic variance by the residual variance ($\sigma_{\varepsilon }^{2}$) in Eq. (4). All analyses were performed in R software, v.4.1.3 (R Core Team 2021).

### Correlation and path analysis

Genotypic correlations were calculated between pairs of traits by fitting a bivariate mixed linear model as follows:

5 $$\left[ {\begin{array}{*{20}c} {y_{ijkl} } \\ {y^{\prime}_{ijkl} } \\ \end{array} } \right] = g_{i} + e_{j} + b_{k} + r_{l} + ge_{ij} + \varepsilon_{ijkl}$$

where *y*_*ijkl*_ is the phenotypic value of the first trait; *y’*_*ijkl*_ is the phenotypic value of the second trait; *g*_*i*_ is the effect of the *i*th genotype; *e*_*j*_ is the effect of the *j*th environment; *ge*_*ij*_ is the effect of the interaction between the *i*th genotype and the *j*th environment; *b*_*k*_ is the effect of the *k*th block; *r*_*l*_ is the effect of the *l*th replicate; and *ε*_*ijkl*_ is the residual error. Bivariate models were fitted with unstructured variance–covariance using the *corh-option* for the genotype, environment and genotype by environment interaction and diagonal variance–covariance for replicate and blocks. In addition to genotypic correlations, Pearson’s product-moment correlations analysis was performed based BLUEs and the results plotted using the GGally package v.2.1.2. Correlation coefficients were classified and interpreted as described by Zou et al. (2003).

**Fig. 1:**
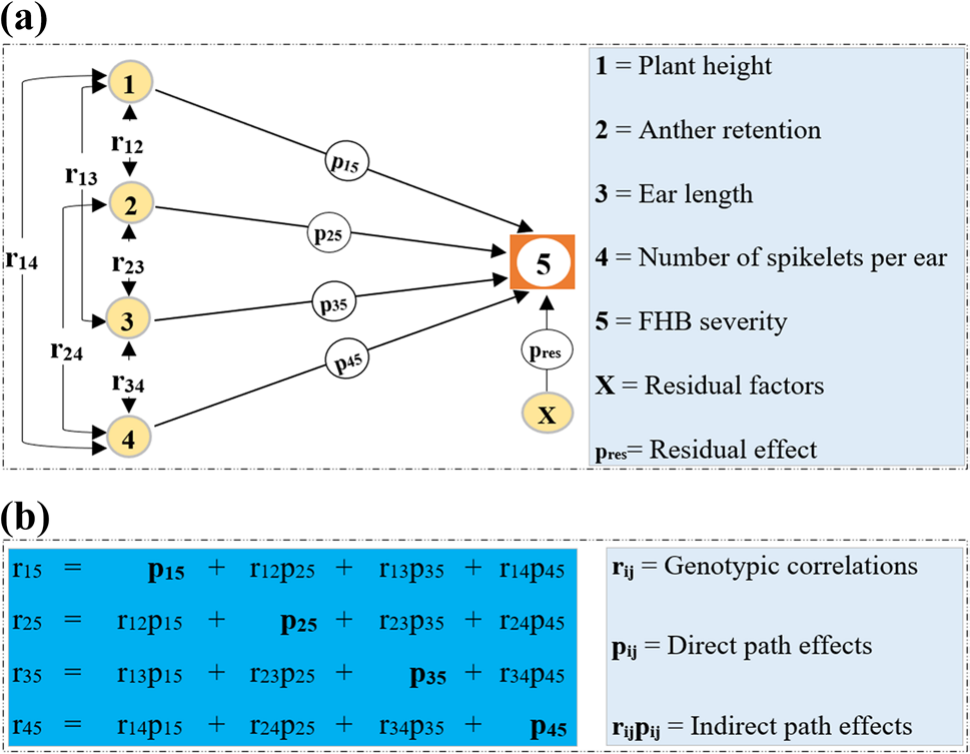
Representation of the nature of the interactions among traits. **a** = path diagram illustrating direct and indirect effects; **b** = simultaneous structured equations matrix showing relationships between correlations and path coefficients. Double-arrowed lines indicate mutual association as measured by genotypic correlations (*r*_*ij*_), and single-arrowed lines represent direct path effects (*p*_*ij*_)

The genotypic correlations were used to perform path analysis between FHB severity, as response variable, and morphological traits such as PH, AR, EL and NS included as explanatory variables in agricolae R package v.1.3–5 (De Mendiburu 2016). The analysis was based on standardized partial regressions which used observed genotypic correlations to estimate direct and indirect path effects as illustrated diagrammatically in detail in Fig. 1 (Wright 1918, 1934; Dewey and Lu 1959). For simplicity, PH, AR, EL and NS were coded as 1, 2, 3, and 4, respectively. The path model was fitted as follows:

6 $$Y\, = \,\hat{\beta }\, X\, + \,\varepsilon$$

where $\hat{\beta }$ is the standardized regression slope, representing the direct effect estimators; *Y* is the vector of genotypic correlations between FHB severity and morphological traits; *X* is the genotypic correlation matrix among morphological traits; and *ε* is a vector of residual errors. To estimate the direct effect of each exploratory variable on FHB severity, we minimized the residual least squares and took its derivative with respect to $\hat{\beta }$, creating a system of normal equations as described by Toebe and Cargnelutti (2013):

7 $$\hat{\beta }\, = \,\left[ {X^{\prime}X} \right]^{ - 1} X^{\prime}Y\, = \,\left[ {\begin{array}{*{20}c} {\begin{array}{*{20}c} {p_{15} } \\ {p_{25} } \\ {p_{35} } \\ \end{array} } \\ {p_{45} } \\ \end{array} } \right]$$

where *X’* and *X*^*−1*^ are the transpose and inverse of the correlation matrix *X*, respectively; *p*_*15*_, *p*_*25*_, *p*_*35*_ and *p*_*45*_ are the direct path effects of PH, AR, EL and NS on FHB severity, respectively. Afterwards, the indirect path effects were estimated using structured equations (Dewey and Lu 1959) linking the correlation coefficients and the direct path effects as follows:

8 $$r_{i5} = p_{i5 } + \sum r_{ik} p_{k5}$$

where *r*_*i5*_ is the genotypic correlation coefficient between the *i*th trait and FHB severity; *p*_*i5*_ is the direct path effect of the *i*th trait on FHB severity; and *∑r*_*ik*_*p*_*k5*_ is the summation of indirect path effects of the *i*th trait on FHB severity via all other *k* traits. We obtained four structured and simultaneous equations which defined a matrix of direct (diagonal elements) and indirect path (off-diagonal elements) effects of morphological traits on FHB severity (Fig. 1b). Coefficient of determination (*R*^*2*^) of the path model was estimated as:

9 $$R^{2} \, = \,r_{15} p_{15} \, + \,r_{25} p_{25} \, + \,r_{35} p_{35} \, + \,r_{45} p_{45}$$

where *r*_15_, *r*_25_, *r*_35_ and *r*_45_ are the genotypic correlations between FHB severity and PH, AR, EL and NS, respectively. The direct effect (*p*_res_) of the residual factors was calculated as the part of the variation of FHB severity which was not explained by PH, AR, EL and NS (Toebe and Cargnelutti 2013). That is,

10 $$p_{{{\text{res}}}} \, = \,\sqrt {1\, - \,R^{2} }$$

The residual factors referred to all other variables which contribute to the variation of FHB severity and were not included in the path model. Similar to the standard multivariate modelling, the existence of high multicollinearity among exploratory variables can lead to significant biases in the estimates of path effects and result in a wrong interpretation of existing causal relationships among the variables (Stage et al. 2010; Toebe and Cargnelutti 2013). Therefore, we investigated the multicollinearity among exploratory variables by calculating the variance inflation factor (VIF). In doing this, a linear regression was fitted for each variable on other variables, and the respective multiple *R*^2^ ($R_{{{\text{mult}}}}^{2}$) was used to calculate VIF as:

11 $${\text{VIF}}\, = \, \frac{1}{{1\, - \,R_{{{\text{mult}}}}^{2} }}$$

Multicollinearity was considered as high, if VIF > 5 (Olivoto et al. 2017).

### Population structure and genome-wide association studies

Polymorphic and high-quality SNP markers obtained after the filtering were used to investigate the population structure of the 401 genotypes, using the *snpdgsPCA()* function available in SNPRelate R package v.1.26 (Zheng 2015). Scatter plots illustrating the structure of the population were built for the first two principal coordinates using the ggplot2 package v.3.3.5 (Wickham et al. 2021).

To dissect the genetic architecture of FHB severity, PH and AR, single genome-wide association study (ST-GWAS) analysis was performed based on all 19,969 SNP markers with MAF ≥ 0.05 to identify significant marker–traits associations (MTAs), using the Bayesian-information and Linkage-disequilibrium Iteratively Nested Keyway (BLINK) method available in the Genomic Association and Prediction Integrated Tool (GAPIT) R package v.3.1.0 (Wang and Zhang 2021). BLINK is a multi-locus GWAS method which was demonstrated to outperform in both speed and statistical power other methods such as the fixed and random model circulating probability unification (FarmCPU) and mixed linear model (MLM) (Huang et al. 2019; Wang and Zhang 2021). In contrary to FarmCPU, BLINK works directly on the markers instead of bins, thereby removing the assumption that causal loci are evenly distributed across the genome. BLINK includes two fixed effects models (FEM_1_ and FEM_2_) and one filtering process, which are performed iteratively (Huang et al. 2019). The filtering process consists in sorting all markers and selecting the first *t* most significant markers referred to as pseudo-quantitative trait nucleotides (QTNs) (based on indicated threshold) that are not in linkage disequilibrium (Pearson’s correlation < 0.7) as covariates. Based on this, the two FEMs can be written as follows:

12 $${\text{FEM}}_{1} \,:\,y_{i } \, = \,S_{i1} b_{1} \, + \,S_{i2} b_{2} \, + \,S_{i3} b_{3} \, + \, \ldots \, + \,S_{ik} b_{k} \, + \,S_{ij} d_{j} \, + \,\varepsilon_{i}$$

13 $${\text{FEM}}_{2} : \, y_{i} \, = \,S_{i1} b_{1} \, + \,S_{i2} b_{2} \, + \,S_{i3} b_{3} \, + \, \ldots \, + \,S_{ik} b_{k} \, + \,\varepsilon_{i}$$

where *y*_*i*_ is the BLUE across environments of the *i*th genotype; *S*_*i1*_, *S*_*i2*_, …, *S*_*ik*_ are the genotype scores of *k* pseudo-QTNs, initiated as an empty set; *b*_*1*_, *b*_*2*_, …, *b*_*k*_ are the respective effects of the pseudo-QTNs; *S*_*ij*_ is the genotype score of the *i*th genotype and *j*th marker; *d*_*j*_ is the corresponding effect of the *j*th marker; and *e*_*i*_ is the residual with *e*_*i* _~ *N(0,* $\sigma_{\varepsilon }^{2}$*)*. In FEM_1_, *S*_*ij*_*d*_*j*_ represents a testing markers term where all remaining markers are tested one at a time. In FEM_2_, all t pseudo-QTNs selected after the filtering are re-examined together using the Bayesian information content (BIC) as an optimization criterion. Afterwards, *k* out of the *t* pseudo-QTNs with the best BIC values are selected and included in FEM_1_ as covariates to test the remaining markers.

As BLINK method does not automatically make a clear separation between major- and small-effect QTL (pseudo-QTNs selected are retested together and some are also removed in FEM_2_), we implemented two ST-GWAS analyses (ST-GWAS_1_ and ST-GWAS_2_) on each of the trait. In ST-GWAS_1_, all markers were included in the analysis, while in ST-GWAS_2_ significant MTAs identified on chromosomes 4D (*Rht-D1*) and 6A (*Rht24*) from ST-GWAS_1_ were used as covariate variable to increase the detection of small-effect markers. In wheat, the use of *Rht* markers as covariate variables in BLINK was reported to increase the number of significant markers (Merrick et al. 2022). Significant MTAs were identified based an exploratory threshold of − log10 (*p* value) = 6 and a Bonferroni-corrected threshold of − log10 (*p* value) = 6.30. The corrected Bonferroni threshold (*p* value) was determined as follows:

14 $$p\;{\text{value}}\, = \,\frac{\alpha }{{{\text{Me}}}}$$

where *α* = 0.01 is the type 1 error and Me is the number of markers included in the analysis. The total proportion of genetic variation (*p*_*G*_) explained by significant markers for each trait was determined as follows (Utz et al. 2000):

15 $$p_{G} \, = \, \frac{{R_{{{\text{adj}}}}^{2} }}{{H^{2} }}$$

where $R_{{{\text{adj}}}}^{{2}}$ is the adjusted *R*^*2*^ and $H^{{2}}$ is the broad-sense heritability of the trait. The adjusted *R*^*2*^ was estimated by fitting a multiple linear regression model as follows:

16 $$y\, = \, {\text{snp}}_{i} \, + \,{\text{snp}}_{j} \, + \, \ldots \, + \, {\text{snp}}_{k} \, + \,\varepsilon$$

where *y* is the BLUEs and snp_*i*_, snp_j_, and snp_*k*_ are the SNP markers identified by ST-GWAS with threshold of snp_*i*_ > snp_*j*_ > … > snp_*k*_ (Gaikpa et al. 2020). Individual *p*_*G*_ values were calculated using the sum of squares (SS) from the analysis of variance of the linear model (Würschum et al. 2015) as:

17 $$p_{G} \, = \, \frac{{{\text{SS}}_{{{\text{snp}}}} }}{{{\text{SS}}_{{{\text{total}}}} \, \times \, H^{2} }} \, \times \,100$$

where SS_snp_ is the sum of squares of individual SNP and SS_total_ is the total sum of squares of the model. The sum of squares was estimated using the SS type II to avoid changes in the estimates due to SNPs order in the model. Additive effects were estimated by fitting individual SNP in the linear model one at a time as described by Gaikpa et al. (2020).

Additionally, pleiotropic loci between FHB severity, PH and AR were investigated by performing a multi-trait GWAS (MT-GWAS) using the multi-trait mixed-model (MTMM) method proposed by Korte et al. (2012). MT-GWAS was implemented in ASRemL-R package v.4.1.0 between FHB severity and PH (FHB vs PH), FHB severity and AR (FHB vs AR) and PH and AR (PH vs AR). For each pair of traits, the MTMM was:

18 $$\left[ {\begin{array}{*{20}c} {y_{1} } \\ {y_{2} } \\ \end{array} } \right]\, = \,\mathop \sum \limits_{i = 1}^{2} S_{i} \mu_{i} \, + \,X\beta \, + \,(X\, + \,S_{i} )\alpha \, + \,\upsilon$$

where *y*_*1*_ and *y*_*2*_ are the phenotypes of the first and second traits, respectively; *S*_*i*_ is a vector of 1 for all values belonging to the *i*th trait; *µ*_*i*_ is a vector of fixed effects including the mean of the *i*th trait; *X* is the vector of SNP markers; *β* is the vector of common effects between the two traits; *α* is the vector of interaction effects; and *υ* is a random variable capturing both the error and genetic random effects such as the kinship matrix (K). To identify causal loci with common effects (COM) as well as opposite/interaction effects (IE) on each pair of traits, we performed three generalized least squares (GLS) F tests (Korte et al. 2012). Firstly, the full model was tested against a null model where *β* = 0 and *α* = 0 in order to identify the combination of both COM and IE loci. Secondly, COM loci were detected by testing the model with *α* = 0 against the null model. Thirdly, the model with *β* = 0 was tested against the full model to identify IE loci. Significant effects were identified based on the same exploratory and Bonferroni-corrected threshold used in ST-GWAS. This helped to further reduce potential false positives due to inflation of *p* values from F tests as found by Merrick et al. (2022). The genotypic correlation between traits pertaining to each pleiotropic locus (*r*_snp_) was estimated by fitting the significant markers as fixed effects in the bivariate model described in Eq. (5). Markers were fitted consecutively one after the other starting from the highest *p* value to the lowest; that is, threshold of snp_*i*_ > snp_*j*_ > … > snp_*k*_. In that order, the genetic correlation attributable to the *j*th SNP was the difference between the correlation of the model after fitting the *i*th SNP and the one detected after the *j*th SNP. Furthermore, the proportion of genetic correlation explained by each pleiotropic locus was calculated as:

19 $$p_{Ci} \, = \,\frac{{r_{{{\text{snpi}}}} }}{{r_{g} }}\, \times \,100$$

where *p*_*Ci*_ is the proportion of genotypic correlation explained by the *i*th SNP and *r*_*g*_ is the total genotypic correlation obtained from Eq. (5).

For both ST-GWAS and MT-GWAS, the genomic relationship matrix was estimated using the kinship algorithm, VanRaden (2008) in GAPIT v.3.1.0. The kinship matrix (K) was fitted as covariate variable in each GWAS model. Quantile–quantile (Q–Q) plots were used to evaluate the power of the model in controlling false positives and negatives. Q–Q plots having a straight line, close to 1:1 on the diagonal, with a sharp upward deviated tail, indicate a good control of both false positives and negatives by the model and confirm the existence of true MTAs (Kaler et al. 2020). Q–Q and Manhattan plots were drawn using the R package CMplot v.4.0.0 (Yin et al. 2021). Moreover, linkage disequilibrium (LD) patterns were also investigated by calculating *R*^2^ values between pairs of significant markers to identify chromosomal positions of unmapped SNP markers. Markers were considered to be in LD when *R*^2 ^≥ 0.70.

### Genomic prediction and marker-assisted selection

Genomic prediction analysis was performed using the mixed linear ridge regression model in the rrBLUP package v.4.6.1 (Endelman 2011). The model (GP_1_) included all markers as random effects and was fitted as follows:

20 $$Y\, = \, \beta \, + \, {\text{Zu}}\, + \,\varepsilon$$

where *Y* is the vector of BLUEs; *β* is a vector of fixed effect such as the grand mean; *Z* is the design matrix; *u* ~ *N(0, A* $\sigma_{\varepsilon }^{2}$*)* is the vector of random markers effects; *A* is a relationship matrix and the residuals are normal with constant variance; and *ε* is a vector of residual errors. GP_1_ model was validated using the fivefold cross-validation approach where the training and validation sets were constituted by splitting the phenotypic and genotypic data into five sets, consisting of 80–81 genotypes each (Gaire et al. 2022). Sampling was repeated 50 times, and GP_1_ model was run for each set to determine the genomic prediction ability (*r*_MG_) and accuracy (*r*_MG/H_) for each of the traits. Genomic prediction ability was the Pearson correlation coefficient between predicted and observed phenotypes for each of the five validation sets (Ould Estaghvirou et al. 2013). Moreover, the prediction accuracy was determined for each validation set as described by Ould Estaghvirou et al. (2013):

21 $$r_{{\text{MG/H}}} { = }\frac{{r{\text{MG}}}}{{\sqrt {H^{{2}} } }}$$

where $H^{2}$ is the broad-sense heritability of the trait. *r*MG and *r*MG/H values were averaged over the five validation sets to obtain the prediction ability and accuracy of each GP_1_ model. Additionally, we evaluated the potential of marker-assisted selection (MAS) using only significant markers from ST-GWAS which explained at least 5% of the genetic variation. MAS prediction was done based on the markers effects deducted from the GP_1_ model, and the prediction accuracy was calculated.

### Estimation of genomic background effect for PH, FHB severity and AR and selection of resistant genotypes with *Rht-D1b*

Based on the ST-GWAS, we evaluated the contribution of GB to FHB severity, PH and AR using the additive effects and estimates of total genetic variation (*p*_*G*_ values) explained by GB and *Rht* markers. *p*_*G*_ values were estimated separately for GB and *Rht* markers by fitting a linear regression model and using the adjusted *R*^2^ as described in Eq. (15). In addition, a second genomic prediction model (GP_2_) was built and the prediction ability (r_MG_) and accuracy (*r*_MG/H_) were evaluated following the fivefold approach. Training and validation sets were the same as described for GP_1_. In GP_2_, significant MTAs for PH on chromosomes 4D and 6A were used as covariates, and the prediction accuracy was estimated for each trait based on small-effect markers only. GP_2_ was fitted as follows:

22 $$Y\, = \,X\beta \, + \,{\text{Zu}}\, + \,\varepsilon$$

where *Y* is the vector of BLUEs; *β* is the vector of fixed effects of the covariates; *X* and *Z* are the design matrices; *u* ~ *N(0, A* $\sigma_{\varepsilon }^{2}$*)* is the vector of random markers effects; *A* is a relationship matrix and the residuals are normal with constant variance; and *ε* is a vector of residual errors. Furthermore, we selected the ten best genotypes with low FHB severity and *Rht-D1b* allele based on the calculation of genetic estimated breeding values (GEBV) using the effects of random markers from the GP_2_ model as:

23 $$Y_{0} = Z_{0} u$$

where *Y*_*0*_ and *Z*_*0*_ are the vectors of GEBV and design matrix of the genotypes, respectively. The correlation between the GEBV-based approach and stacking of additive effects (SAE) of GB markers from ST-GWAS was also determined in order to check the relative effectiveness of GWAS for exploiting genomic background. SAE was calculated only for GB markers which explained at least 5% of genetic variation.

## Results

### Considerable genetic variation was found for FHB severity and morphological traits

Genetic coefficients of variation (CV_*G*_) were low for DTF, HS, EL, NS and ED and moderate to high for PH, FHB severity and AR (Table 1). The higher CV_G_ observed for AR was due to the considerable existing genetic variation among the genotypes. The coefficient of variation due to error (CV_*ε*_) was low for all traits (Table 1). Genetic variances ranged from 0.03 to 6.9 for DTF, HS, EL, NS and ED and were higher (97.4–489.4) for PH, FHB and AR. The genotype–environment interaction (GEI) was significant and generally lower than the genetic variance. Depending on the trait, GEI variance was 1.5–22.2-fold lower than the genetic variance. Broad-sense heritability estimates were very high (> 0.9) for all traits.

**Table 1:** Descriptive statistics, variance components and broad-sense heritability estimates for all traits

Trait	Unit	Mean	Range	CV_*G*_ (%)	CV_*ε*_ (%)	Variance components			*H*^2^
						$\sigma_{G}^{2}$	$\sigma_{{{\text{GE}}}}^{{2}}$	$\sigma_{\epsilon}^{2}$	
PH	cm	91.40	52.42	10.80	3.31	97.40	5.89	9.13	0.97
FHB	%	37.78	58.29	29.53	11.81	124.43	35.58	19.90	0.92
DTF	Days	43.58	7.60	3.49	1.56	2.31	0.31	0.46	0.91
HS	BBCH stage	53.41	12.64	4.93	2.26	6.94	1.41	1.46	0.94
AR	%	55.72	89.10	39.70	11.41	489.39	80.70	40.43	0.95
EL	cm	10.55	5.40	8.26	3.79	0.76	0.06	0.16	0.95
NS	Spikelets ear^−1^	22.46	8.39	6.30	1.99	2.00	0.09	0.20	0.97
ED	Spikelets cm^−1^	2.15	1.28	8.06	0.00	0.03	0.02	0.00	0.96

*PH* plant height, *FHB* Fusarium head blight severity, *DTF*  days to flowering, *HS* heading stage, *AR* anther retention, EL  ear length, NS  number of spikelets per ear, *ED*   ear density, *CV*_*G*_, *CV*_*ε*_ genetic and error coefficient of variation, respectively, $\sigma_{G}^{2}$, $\sigma_{{{\text{GE}}}}^{{2}}$ and $\sigma_{\epsilon}^{2}$ refer to genotypic and genotype × environment interaction and residual variances, respectively, *H*^2^ = broad-sense heritability. All variance components were significant at *p*  <  0.001

### Correlations and path coefficients depict the contribution of morphological traits to FHB severity

**Fig. 2:**
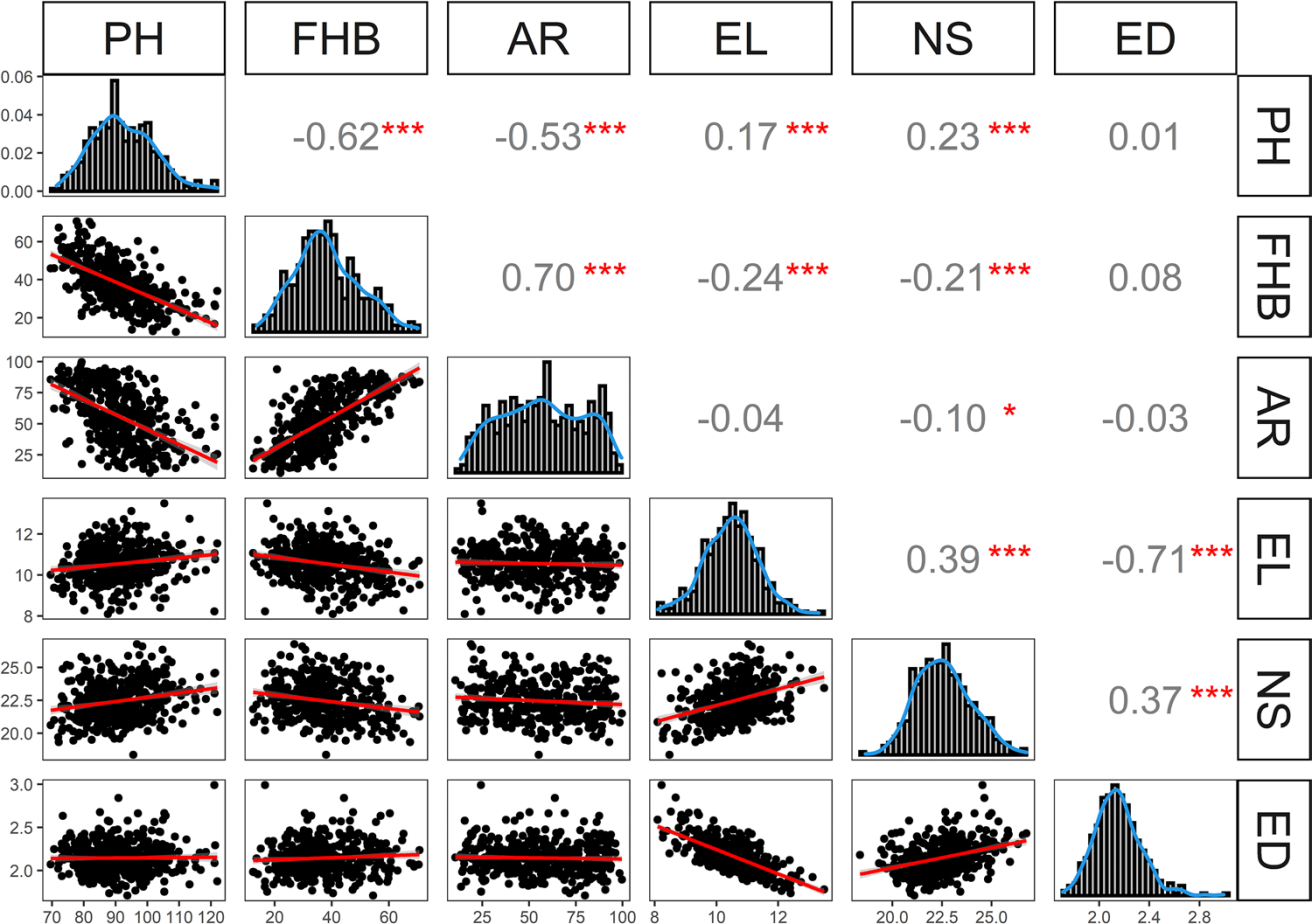
Pearson’s product-moment correlation analysis among traits *, **, ***significant at *p*  < 0.05, 0.01 and 0.001, respectively. PH = plant height, FHB = FHB severity, AR = anther retention, EL = ear length, NS = number of spikelets per ear, ED = ear density.

Density plots of BLUEs across environments showed nearly normal distribution for all traits (Fig. 2). Linear relationships with significant phenotypic correlations were observed among traits. FHB severity was positively and highly correlated with AR (*r*_*p*_ = 0.70) and negatively and moderately correlated with PH (*r*_*p*_ = − 0.62). The outlying point in the scatter plot FHB severity/AR belongs to the cleistogamic cultivar “Anapolis” that has a very high AR (99.6%) and a low FHB severity (20.9%). PH was negatively and moderately correlated with AR (*r*_*p*_ = − 0.53), while NS showed positive and low correlation with EL (*r*_*p*_ = 0.39) and ED (*r*_*p*_ = 0.37). Highly negative phenotypic correlations were detected between EL and ED (*r*_*p*_ = − 0.71) (Fig. 2). Phenotypic correlations between FHB severity and EL and NS were significant, but low. At the genetic level, the correlations were negative and moderate between FHB severity and PH (*r*_*g*_ = − 0.64), PH and AR (*r*_*g*_ = − 0.55) and negatively high between ED and EL (*r*_*g*_ = − 0.70). Genotypic correlations were positively high between FHB severity and AR (*r*_*g*_ = 0.74) and low between NS with EL (*r*_*p*_ = 0.38) and ED (*r*_*p*_ = 0.37) (Table 2).

**Table 2:** Genotypic correlation coefficients among traits, excluding days to flowering and heading stages that both were used as covariates (*r*_*g*_, above diagonal), and corresponding standard errors (SE, below diagonal)

Traits	FHB severity	PH	AR	EL	NS	ED
FHB severity		− 0.64	0.74	− 0.30	− 0.24	0.09
PH	0.03		− 0.55	0.18	0.24	0.02
AR	0.02	0.04		− 0.05	− 0.11	− 0.03
EL	0.05	0.05	0.05		0.38	− 0.70
NS	0.05	0.04	0.05	0.04		0.37
ED	0.05	0.05	0.05	0.02	0.04	

*PH* plant height, *FHB*  Fusarium head blight, *AR* anther retention, *EL* ear length, *NS* number of spikelets per ear, *ED* ear density. All correlation coefficients were significant at *p* < 0.001

The causal system formed by PH, AR, EL and NS explained 67.04% of the variation of FHB severity (Table 3). Path effect due to residual factors was 0.56. The direct effect of AR (0.57) was 3.3-fold higher than its indirect paths and similar to the residual effect. The indirect effect of PH via AR (− 0.31) was slightly higher than its direct effect (− 0.28), but both effects were lower than the residual effect. Direct and indirect effects of EL were considerably lower than the residual effect, while effects of NS were not significant. Inflation due to multicollinearity among PH, AR, EL and NS was negligible (VIF < 2), showing that estimates of direct and indirect effects and coefficient of determination were accurate (Table 3).

**Table 3:** Direct and indirect path effects of morphological traits on FHB severity

Traits	*r*_*g*_	VIF	Direct effects	Indirect effects			
				PH	AR	EL	NS
PH	− 0.64	1.47	− 0.28**		− 0.31	− 0.04	− 0.01
AR	0.74	1.40	0.57***	0.15		0.01	0.01
EL	− 0.30	1.19	− 0.21*	− 0.05	− 0.03		− 0.01
NS	− 0.24	1.21	− 0.03	− 0.07	− 0.06	− 0.08	
Residual effect (*β*_res_) = 0.56				Coefficient of determination (*R*^2^) = 0.67			

*PH *plant height, *AR* anther retention, *EL* ear length, *NS* number of spikelets per ear, *FHB* Fusarium head blight; *r*_*g*_  genotypic correlation, *VIF*  variance inflation factor

*, **, ***Significant at *p*  < 0.05, 0.01 and 0.001, respectively

### Several marker-trait associations (MTAs) were detected by genome-wide association (GWA) studies

**Fig. 3:**
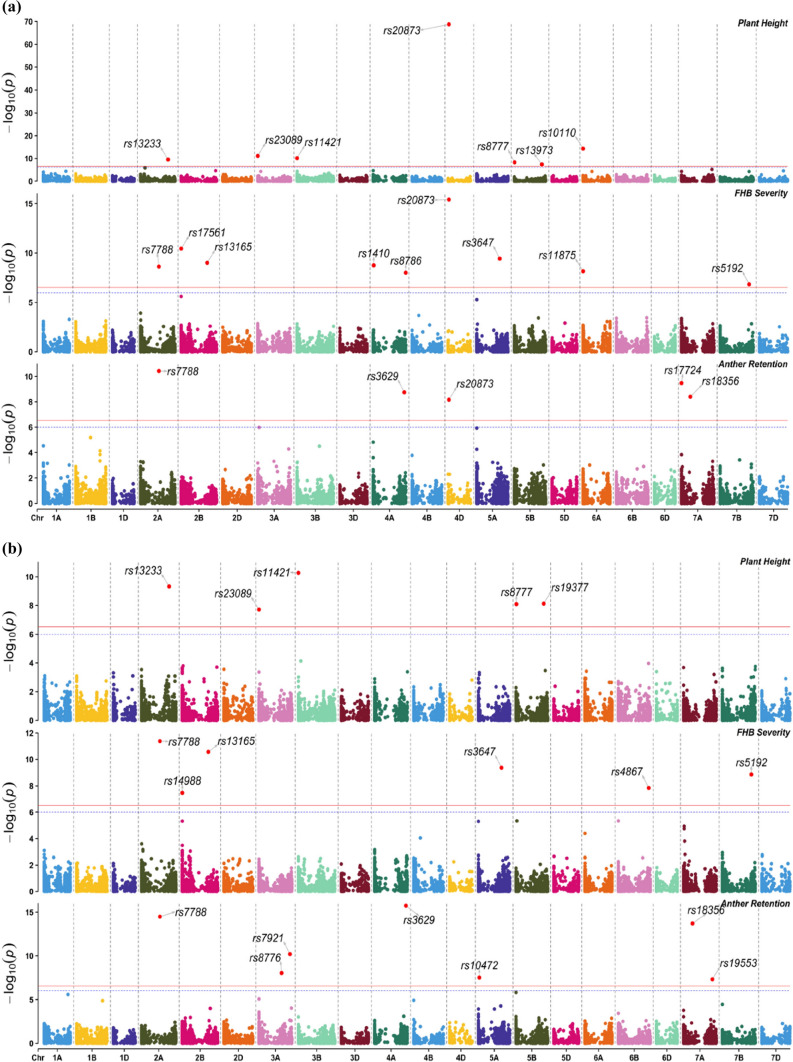
Manhattan plots highlighting significant marker–trait associations (MTAs) for single-trait genome-wide association studies (ST-GWAS): **a** = ST-GWAS_1_ including all markers and **b** = ST-GWAS_2_ without markers linked to plant height on chromosomes 4D (*Rht-D1*) and 6A (*Rht24*). The blue dotted line corresponds to an exploratory threshold of − Log 10(*p*) = 6, while the red plain line represents the Bonferroni-corrected threshold cut-off of alpha = 0.01 (color figure online)

The first two principal coordinates (PCs) indicated that the 401 genotypes included in the study were not structured (Fig. S1). Therefore, we corrected in the GWAS for relatedness among genotypes using the kinship matrix and did not include PCs. The ST-GWAS was performed to depict the genetic architecture of FHB severity, PH and AR (Table S2). Considering all traits, a total of 25 significant MTAs were identified, of which seven were specific to ST-GWAS_1_, seven to ST-GWAS_2_ and eleven common to both models (Fig. 3a, b). The use of *Rht* markers as covariate variables in ST-GWAS_2_ identified additional small-effect MTAs for all traits. Particularly, the number of small-effect MTAs increased from four to seven for AR, while two new MTAs were identified for FHB severity. In total, eight, eleven and nine MTAs were identified for PH, FHB severity and AR, respectively (Table S2). One MTA was common to PH and FHB severity, one to PH and AR and two to FHB severity and AR. MTAs explained 69.7, 60.7 and 44.2% of total genetic variation for PH, FHB severity and AR, respectively. Markers *rs20873* on chromosome 4D linked to *Rht-D1* and *rs10110* on chromosome 6A linked to *Rht24* were major MTAs for PH (Fig. 3a, b), which explained 45.2 and 10.8% of genetic variation, respectively. In addition, *rs20873* conveyed the largest reduction effect of − 7.02 cm on PH. For FHB severity, major MTAs were *rs20873*, *rs5192* on chromosome 7B and *rs3647* on chromosome 5A. Likewise, *rs20873* was the major MTA identified for AR, explaining 22.7% of genetic variation. *rs20873* increased FHB severity and AR by about 6 and 12%, respectively. The false discovery rates were very low (FDR < 0.05) for identified MTAs.

The MT-GWAS identified a total of six pleiotropic loci distributed on chromosomes 2A, 4D, 5A, 6B and 7B. All six were detected between FHB severity and PH (Table 4a), four between FHB and AR (Table 4b) and three between PH and AR (Table 4c). In comparison with the ST-GWAS from BLINK, the marginal trait from the MT-GWAS identified three additional MTAs on chromosomes 4D, 6B and 5A for FHB severity, one on chromosome 5A for PH and one on chromosome 4D for AR. However, the MT-GWAS identified three MTAs for FHB severity, one for PH and three for AR, which were also detected by ST-GWAS. Three out of the six MTAs detected for FHB versus PH exhibited positive contribution to the genotypic correlation and thus exerted common effects on FHB severity and PH (Table 4a). Inversely, the remaining three MTAs showed negative genotypic correlations, revealing their opposite effects on both traits. Marker *rs9660* on chromosome 4D was associated with FHB severity only, but it exerted an opposite effect on the two traits. Similarly, all common effects MTAs were associated with FHB severity only. Common effect MTAs explained 6.3–26.6% of the genotypic correlation between FHB severity and PH, while 3.1–6.3% of the correlation was attributable to interaction effects MTAs (Table 4a). For FHB versus AR, all four MTAs identified by the full model had common effects with 1.4–14.9% of the genotypic correlation explained between FHB severity and AR (Table 4b). Although marker rs5192 on chromosome 7B was specific to FHB severity, it had a common effect on both traits. For PH versus AR, two out of the three MTAs identified by the full model had opposite effects with 5.5–56.4% of the genotypic correlation explained between the two traits (Table 4c). Marker rs7788 that was specific to AR had a common effect on both PH and AR with 10.9% of the genotypic correlation explained.

**Table 4:** SNP markers associated with common (COM), interaction effects (IE) and the full model (FULL) on plant height (PH), FHB severity and anther retention (AR) from MT-GWAS

Marker	Chr	Pos	UA/FA	FAF	COM	IE	FULL	*Y*_1_	*Y*_2_	*r*_snp_	*p*_C_ (%)
*a. FHB severity (Y*_*1*_*) versus PH (Y*_*2*_*)*											
rs20873	4D	19.19	G/T	0.49	2.00	**29.08**	**29.37**	**30.21**	**16.75**	− 0.17	26.56
rs9660	4D	195.24	G/A	0.15	0.75	**9.13**	**8.40**	**7.92**	1.20	− 0.04	6.25
rs9869	6B	459.72	T/G	0.22	**7.09**	2.52	**8.15**	**6.92**	0.02	0.04	6.25
rs22296	5A	681.46	T/C	0.40	1.45	**6.47**	**6.61**	**7.45**	**7.01**	− 0.05	7.81
rs5192	7B	661.18	A/G	0.66	**6.00**	1.54	**6.00**	**7.00**	2.44	0.02	3.12
rs7788	2A	419.17	A/G	0.36	**6.39**	0.72	**6.00**	**10.82**	2.50	0.04	6.25
*b. FHB severity (Y1) versus AR (Y*_*2*_*)*											
rs20873	4D	19.19	G/T	0.49	**17.60**	0.47	**16.75**	**16.75**	**12.62**	0.11	14.86
rs9660	4D	195.24	G/A	0.15	**8.38**	0.34	**7.62**	**7.92**	**6.08**	0.03	4.05
rs7788	2A	419.17	A/G	0.36	**6.00**	0.57	**6.55**	**7.19**	**7.19**	0.01	1.35
rs5192	7B	661.18	A/G	0.66	**7.00**	2.71	**6.20**	**6.44**	0.70	0.03	4.05
*c. PH (Y*_*1*_*) versus AR (Y*_*2*_*)*											
rs20873	4D	19.19	G/T	0.49	2.65	**30.42**	**31.31**	**30.21**	**12.62**	− 0.31	56.36
rs22296	5A	681.46	T/C	0.40	1.67	**6.50**	**6.83**	**7.45**	2.11	− 0.03	5.45
rs7788	2A	661.18	A/G	0.36	**6.11**	1.31	**6.14**	0.82	**8.19**	0.06	10.91

*Chr* chromosome, *Pos* physical position on the wheat reference genome RefSeq v.2.1, *UA/FA* unfavourable allele /favourable allele, *FAF* favourable allele frequency, *Y*_1_ = first trait, *Y*_2_ = second trait, FHB = Fusarium head blight, *r*_snp_ = genotypic correlation between *Y*_1_ and *Y*_2_ pertaining to each marker, *p*_*C*_ = proportion of genotypic correlation explained by each marker. Values highlighted in bold are negative logarithm of *p* value [− Log 10(*p*)] which were higher than the Bonferroni-corrected threshold cut-off of 6

In both ST-GWAS and MT-GWAS, the inspection of Q–Q plots of the two GWAS revealed a good control of false positives and negatives, demonstrating the existence of true MTAs controlling the genetic architecture of each trait as well as their interactions (Figs. S2–S5). The linkage disequilibrium analysis revealed strong linkage (*R*^2^ = 0.9) between markers *rs19377* and *rs13973* located on chromosome 5B for PH (Fig. S6). Very low *R*^2^ values were observed among all other MTAs.

### Significant differences were observed among groups of genotypes

**Fig. 4:**
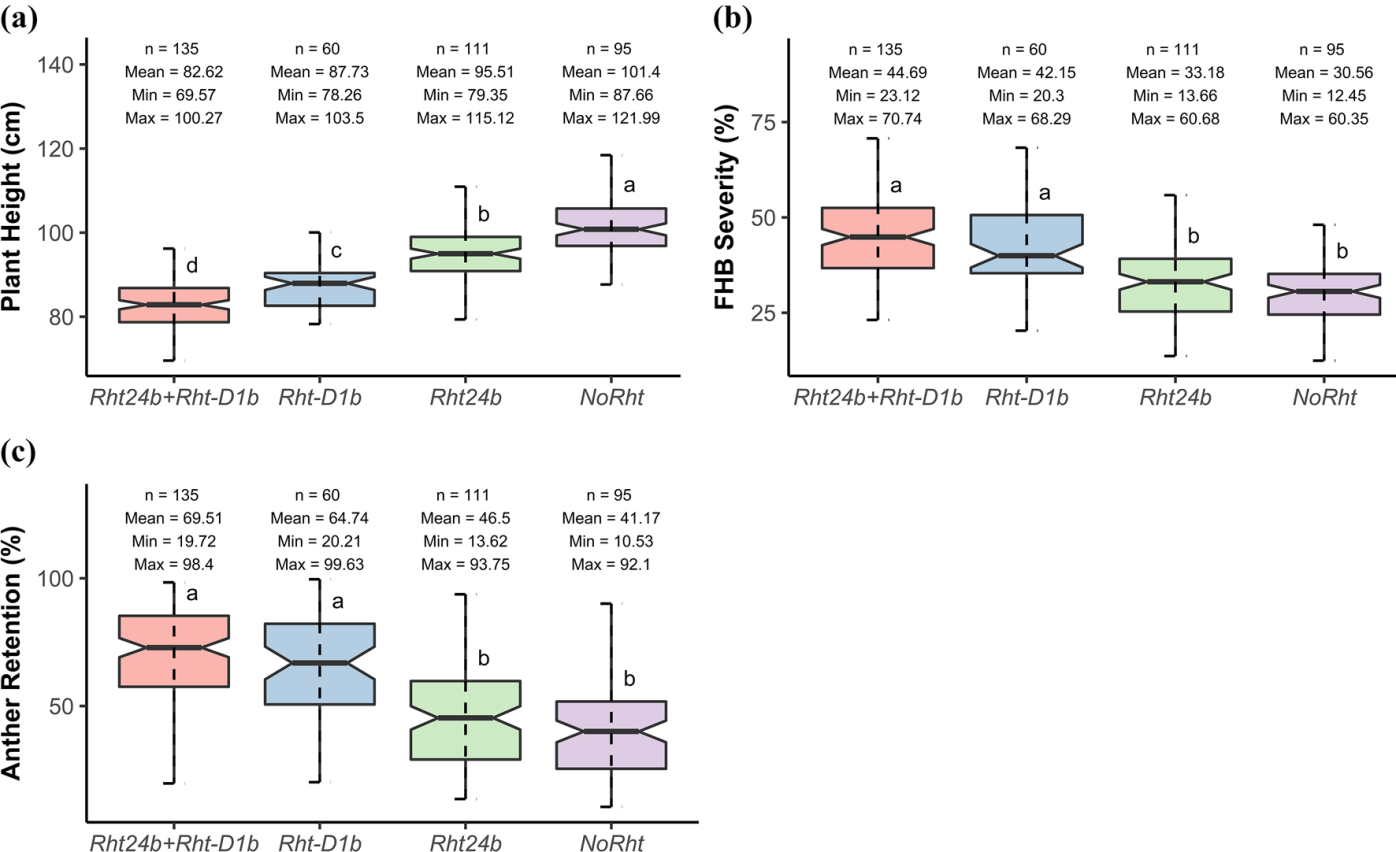
Boxplots showing variation of traits among *Rht* groups. (**a**) = plant height, (**b**) = FHB severity, and (**c**) = anther retention. n = number of genotypes, Min = minimum, Max = maximum. Boxplots with the same superscripts are statistically not significant at *p* < 0.05

NoRht, *Rht24b*, *Rht-D1b* and *Rht24b* + *Rht-D1b* represented, respectively, 23.7, 27.7, 14.9 and 33.7% of our materials (Table 5). Furthermore, considerable genetic variation was observed within each group for PH, FHB severity and AR (Table 5). For PH, the genetic variance was 2.5–6.1-fold higher in NoRht compared with other groups. The lowest genetic variance was observed in *Rht-D1b* for PH. However, in FHB severity, the genetic variance was 1.3–1.3-fold higher in *Rht-D1b* than in NoRht and *Rht24b*. The genetic variance was also 1.5–1.6-fold higher in *Rht24b* + *Rht-D1b* compared with NoRht and *Rht24b* which had similar variation for FHB severity. Genetic variance was high and similar among groups for AR. Genotype–environment interactions were significant and relatively low for the three traits in all groups. Comparative analysis among the four groups revealed statistically significant differences for all traits (Fig. 4). Genotypes were on average shorter in *Rht24b* + *Rht-D1b* and taller in NoRht. In *Rht24b*, genotypes were taller than those in *Rht-D1b* and *Rht24b* + *Rht-D1b*. Contrary to PH, FHB severity was on average lower in NoRht and *Rht24b* than in *Rht-D1b* and *Rht24b* + *Rht-D1b*. FHB severity was statistically identical between NoRht and *Rht24b*, and *Rht-D1b* and *Rht24b* + *Rht-D1b*, respectively. Similar to FHB severity, the average AR was lower in NoRht and *Rht24b* compared with *Rht-D1b* and *Rht24b* + *Rht-D1b*, respectively. No significant difference was found for AR between NoRht and *Rht24b*, as well as *Rht-D1b* and *Rht24b* + *Rht-D1b*. The reduction effect of *Rht* alleles on PH was moderately correlated with FHB severity and AR. Highly negative reduction effect was associated with high FHB severity and AR.

**Table 5:** Genotype grouping using *Rht* markers and within-group genotypic ($\sigma_{G}^{2}$) and genotype × environment interaction ($\sigma_{{{\text{GE}}}}^{{2}}$) variances

Group	Size	PH (cm)		FHB severity (%)		AR (%)	
		$\sigma_{G}^{2}$	$\sigma_{{{\text{GE}}}}^{2}$	$\sigma_{G}^{2}$	$\sigma_{{{\text{GE}}}}^{2}$	$\sigma_{G}^{2}$	$\sigma_{{{\text{GE}}}}^{2}$
No*Rht*	95	212.55	9.88	103.45	31.44	531.18	96.74
*Rht24b*	111	84.57	8.63	100.55	34.04	448.51	82.80
*Rht-D1b*	60	34.65	2.64	131.26	35.69	449.92	80.42
*Rht24* + *Rht-D1b*	135	71.96	2.58	160.05	39.66	512.41	67.74

*PH *plant height, *FHB* Fusarium head blight, *AR* anther retention. All variances were significant at *p* < 0.001

### Genomic prediction (GP) exhibited high prediction ability (rMG) and accuracy (rMG/H) for FHB severity, PH and AR

**Fig. 5:**
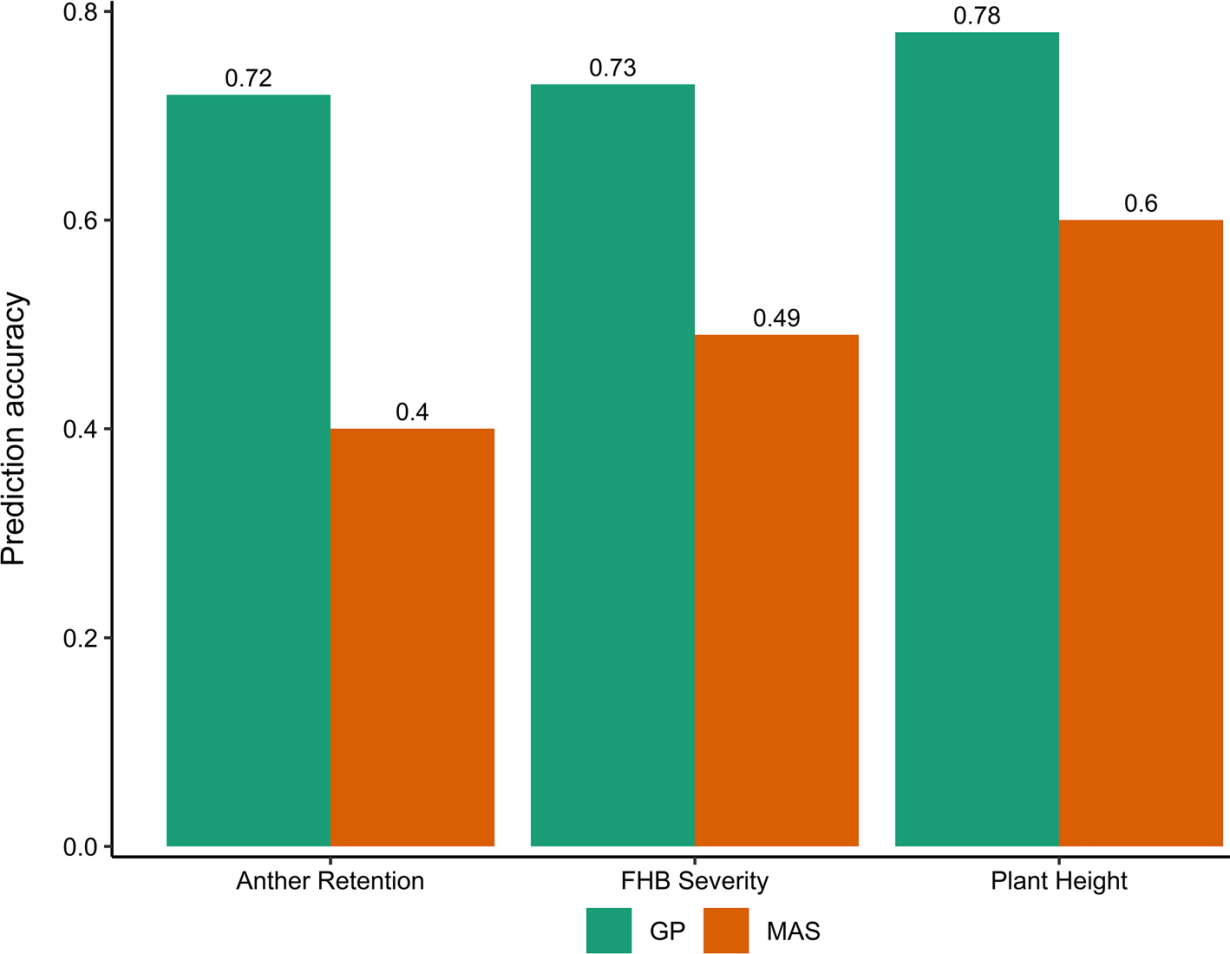
Comparison of prediction accuracies of genomic prediction (GP) and marker-assisted selection (MAS)

Genomic prediction ability and accuracy averaged over the five validation sets were greater than 0.7 depending on the trait (Table S3). FHB severity and AR showed similar and lower prediction accuracy than PH. Genotypes with *Rht-D1b* and *Rht24b* alleles were distributed across all the validation sets, showing a good representation of the genetic variation in each set (Table S4). This guaranteed the accurate estimation of both r_MG_ and r_MG/H_, resulting in low standard errors of the estimates across validation sets (Table S3). In contrary, the prediction accuracy of marker-assisted selection based on SNP markers with *p*_*G*_ ≥ 5% was moderate for all traits, with the lowest value (0.4) observed in AR (Fig. 5). GP_1_ including all available markers increased the prediction accuracy by about 0.2, 0.3 and 0.2 for FHB severity, AR and PH, respectively.

### Genomic background affects PH, FHB severity and AR

**Fig. 6:**
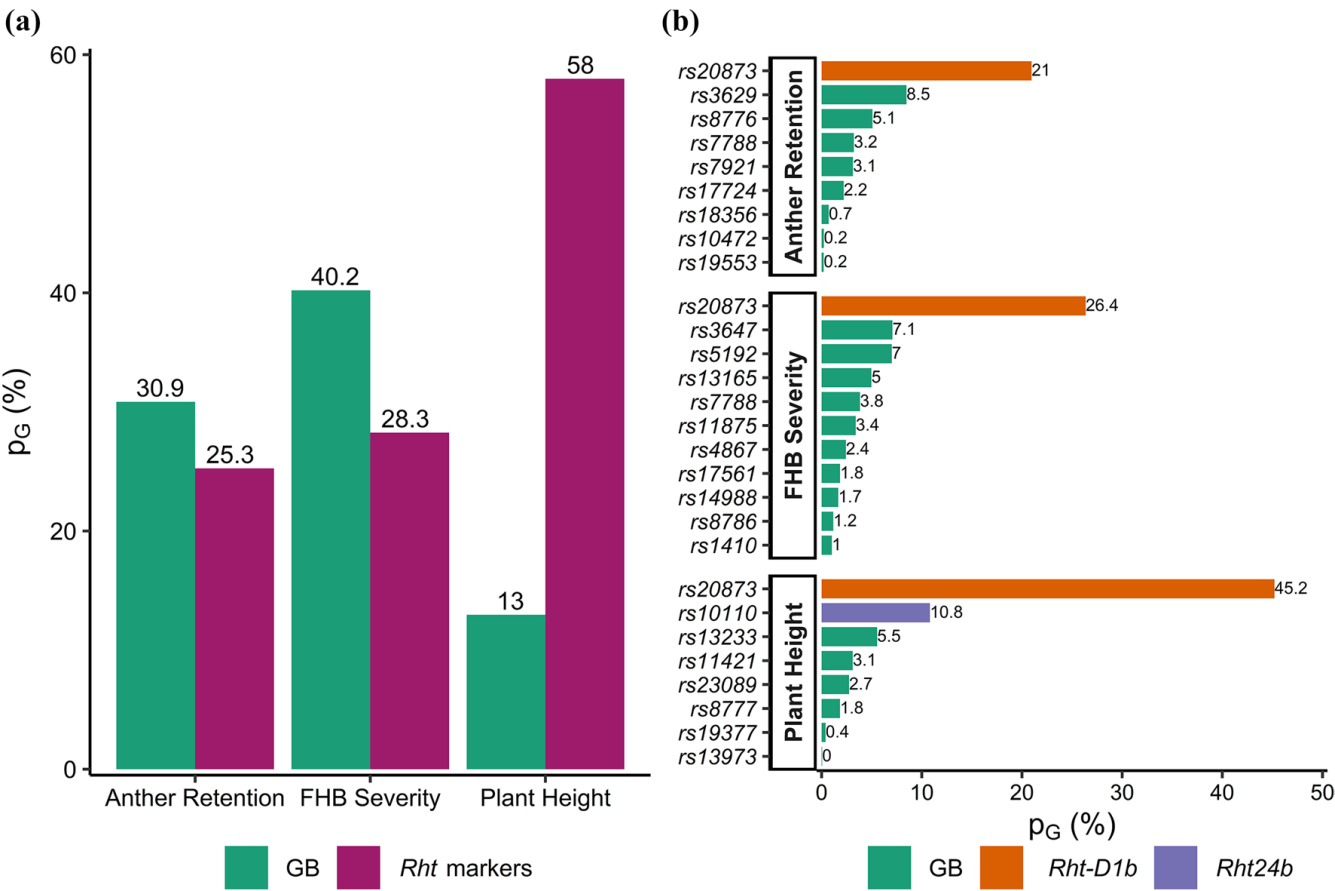
Contribution of *Rht* genes and genomic background (GB) markers to plant height, FHB severity and anther retention. **a = **total genetic variation explained by groups of markers, and **b** = Genetic variation explained by individual markers

**Fig. 7:**
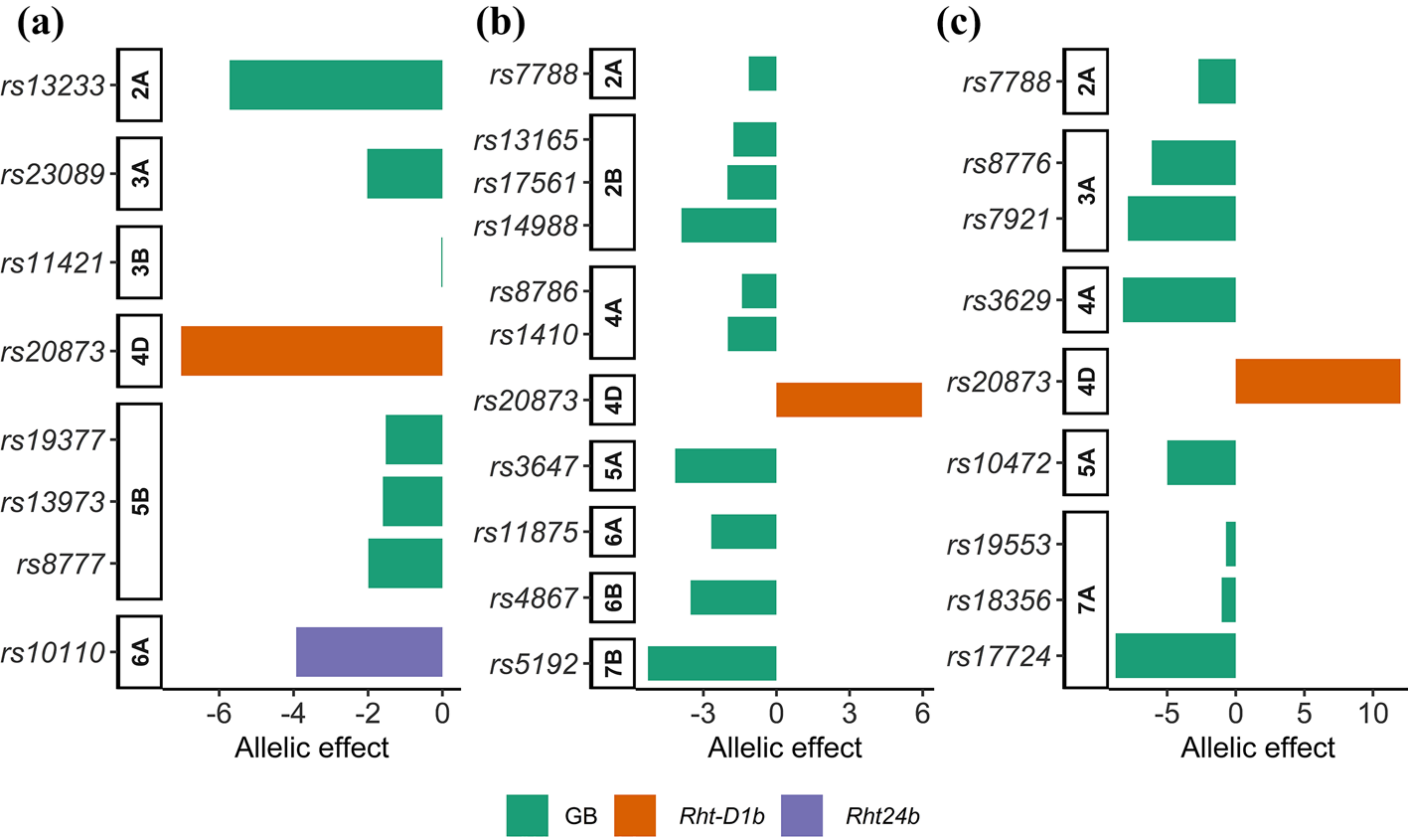
Comparison of additive effects of *Rht* alleles and reduction effects of alleles by genomic background (GB) markers on: **a** = plant height, **b** = FHB severity and **c** = anther retention

The contribution of GB to the genetic variation was different among the three traits (Fig. 6). The total proportion of genetic variation explained by GB markers was higher for FHB severity than PH and AR (Fig. 6a). Contrary to PH, GB markers had higher contribution to the genetic variation of FHB severity and AR than *Rht* markers (Fig. 6a). Ten, eight and six GB markers were found for FHB severity, AR and PH, respectively (Fig. 6b). Non-*Rht* markers which explained at least 5% of the genetic variation were *rs5192*, *rs3647* and *rs13165* for FHB severity, *rs3629* and *rs8776* for AR and *rs13233* for PH (Fig. 6b). Reduction effects exerted by GB markers were relatively high for all traits (Fig. 7a–c). The cumulative reduction effect (CRE) calculated from markers with *p*_*G*_ ≥ 5% was − 5.7 cm, − 11.2% and − 14.3% for PH (Fig. 7a), FHB severity (Fig. 7b) and AR (Fig. 7c), respectively. CRE was about twofold lower than the additive effects of *Rht* markers for PH. Inversely, the CRE exerted by GB markers on FHB severity and AR was 1.3–1.9-fold higher than the increase effect of *Rht* markers (Fig. 7b, c). Results of the second genomic prediction (GP_2_) implemented using *Rht* markers as covariates showed moderate prediction accuracy for the three traits (Table S5). This showed that the estimated contribution of GB was higher in the three traits with GP_2_ compared with GWAS. The highest accuracy was observed for AR, while the lowest value was obtained in PH, confirming the trend revealed by the GWAS. Standard errors were also low for GP_2_, indicating a high consistency of the prediction accuracy among the five validation sets.

### Genotypes with low FHB severity in the presence of *Rht-D1b* were selected using genomic background

**Fig. 8:**
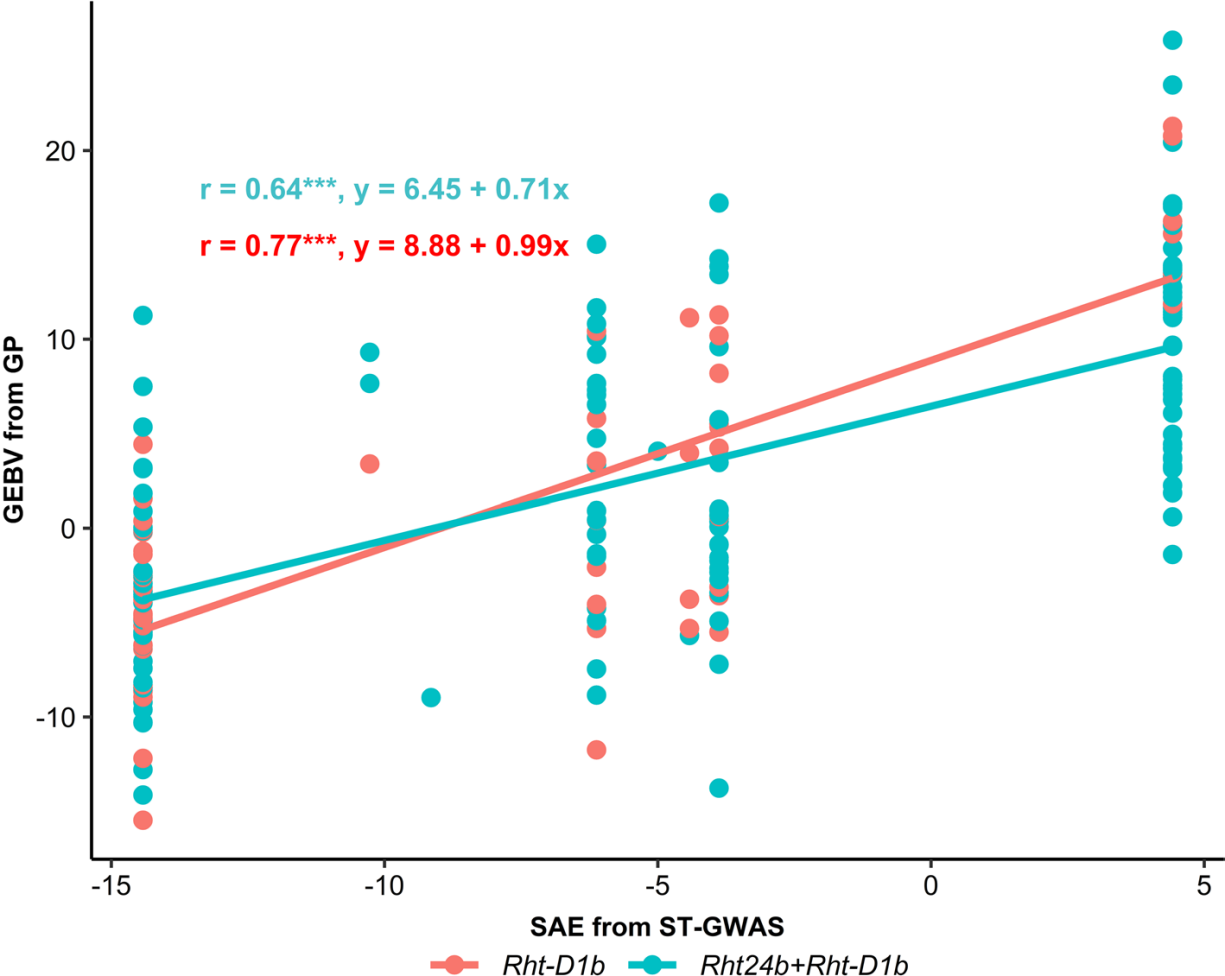
Scatter plot showing the strength of relationship between stacking of additive effects (SAE) of genomic background (GB) markers from single-trait genome-wide association study (ST-GWAS) and genomic estimated breeding values (GEBV) from genomic prediction (GP). SAE was estimated based on GB markers with *p*_*G*_ ≥ 5%. ***significant at *p* < 0.001

Depending on *Rht* groups, GEBV from GP was positively correlated (*r* = 0.64–0.77) with stacking of additive effects (SAE) of GB markers with p_G_ ≥ 5% (Fig. 8). However, several genotypes with same SAE (i.e. − 14.42) exhibited different GEBV. The phenotypic performances of the ten best genotypes selected based on GEBV from the genomic prediction (GP_2_) in *Rht-D1b* and *Rht24b* + *Rht-D1b* groups are summarized in Table 6. The negativity and size of the effects of GB markers as measured by GEBV describe the degree of resistance GB for FHB severity. Thus, highly negative GEBV for FHB severity indicates higher resistance GB, whereas positive GEBV indicates low GB or susceptibility GB. Genotypes with the lowest observed FHB severity exhibited negatively high GEBV for FHB severity carrying *Rht-D1b* allele only (e.g. Faktor and Anapolis), or the combination *Rht24b* + *Rht-D1b* alleles (e.g. Kranich and Kamerad) (Table 6). More resistant genotypes within each *Rht* group also showed negatively high GEBV with lower observed AR, except Anapolis which had a highly positive GEBV for AR and the highest observed AR at all. Taller genotypes had positive GEBV for PH.

**Table 6:** Ten best semi-dwarf genotypes with the highest resistance genomic background (GB) and low FHB severity

Genotype	*Rht* group	FHB severity (%)		AR (%)		PH (cm)	
		GB	BLUE	GB	BLUE	GB	BLUE
Faktor	*Rht-D1b*	− 15.48	20.30	− 38.98	20.21	10.34	100.05
Anapolis	*Rht-D1b*	− 11.75	20.86	30.82	99.63	− 5.75	79.30
Kranich	*Rht24b* + *Rht-D1b*	− 12.79	23.12	− 11.10	48.67	− 2.28	82.85
Kamerad	*Rht24b* + *Rht-D1b*	− 10.28	25.40	8.32	72.87	− 5.26	80.70
Toras	*Rht24b* + *Rht-D1b*	− 10.01	27.66	− 19.33	48.82	3.16	89.46
Esket	*Rht24b* + *Rht-D1b*	− 9.31	28.09	− 9.35	55.69	2.68	87.22
Opal	*Rht24b* + *Rht-D1b*	− 9.22	28.09	− 9.50	51.86	4.17	88.68
Pamier	*Rht24b* + *Rht-D1b*	− 9.55	29.34	− 5.15	58.25	− 2.63	81.77
Mikon	*Rht-D1b*	− 9.20	29.69	− 34.97	23.39	9.02	92.92
Profilus	*Rht24b* + *Rht-D1b*	− 9.61	30.45	− 1.46	50.52	− 2.07	81.15

*AR* anther retention, *FHB *Fusarium head blight, *PH *plant height

## Discussion

Through a combined correlation and path analysis, we provided a first insight into the nature and magnitude of the complex interactions between FHB resistance and morphological traits including plant height and anther retention. ST-GWAS, MT-GWAS and genomic prediction were implemented to depict the genetic architecture of FHB severity, anther retention and plant height and understand the effect of alternative *Rht* genes on FHB resistance. Additionally, multi-trait GWAS was conducted to identify positively and negatively pleiotropic loci controlling complex interactions among traits. The ST-GWAS and genomic prediction helped to evaluate for the first time the contribution of genomic background to each trait and its potential to improve FHB resistance in wheat genotypes with semi-dwarfing allele *Rht-D1b*.

### Existence of high path effects set criteria for effective indirect selection for FHB resistance

FHB severity exhibited high positive correlation with anther retention and moderate negative correlation with plant height at both phenotypic and genotypic levels. Similar observations were reported by Buerstmayr and Buerstmayr (2015); Steiner et al. (2019b) and Ruan et al. (2020). Buerstmayr and Buerstmayr (2015) reported that FHB severity was positively correlated with anther retention (0.63) and negatively with plant height (− 0.39). Previous investigations on anther extrusion, the opposite of anther retention, also highlighted negative correlation of − 0.45 to − 0.64 (Lu et al. 2013) and − 0.55 to − 0.74 (Xu et al. 2020). Recently, Nannuru et al. (2022) also reported negative moderate correlations between FHB severity with anther extrusion (− 0.48) and plant height (− 0.43) in Nordic spring wheat. Our results confirmed that anther retention was negatively correlated with plant height, indicating the existence of shared genetic control between the two traits in winter wheat in contrary to Nannuru et al. (2022) who found a lower correlation (0.16) between plant height and anther extrusion. The differences in the strength of the correlations across studies are an evidence that interactions among the traits are also affected by environment and breeding material characterized by diverse genetic factors.

The causal system formed by morphological traits including anther retention and plant height explained up to 67% of the phenotypic variation of FHB severity in this study. This confirms the importance of morphological traits in the passive resistance mechanism against FHB disease in winter wheat (Mesterházy 1995; Buerstmayr et al. 2020; Xu et al. 2020). The effect due to residual factors was high (0.56) and can be attributed to specific unshared local genetic factors which had considerable influence on FHB severity. The existence of high direct path of 0.57 between FHB severity and anther retention indicates that any increase by 1% in the standard deviation of anther retention implies a direct increase of 0.57% in the standard deviation of FHB severity independently from other traits. This finding exhibits anther retention as a major indicator trait to be included in multiple-trait breeding strategies to aim for FHB-resistant cultivar development in wheat. Particularly, when the breeder’s interest is not in other traits, an indirect selection using anther retention is likely to yield FHB-resistant cultivars (Fernandes et al. 2018; Moreno-Amores et al. 2020). However, path effects between plant height and FHB severity were lower than the residual and the direct effect of anther retention, indicating that plant height as a sole trait does not have considerable contribution to the variation of FHB severity. It is worth noting that the indirect path effect of plant height via anther retention was higher than its direct effect on FHB severity, and this can be explained by the association between plant height and anther retention and underlying genetic factors. A decrease of 1 cm in the standard deviation of plant height in a cultivar would cause a direct increase of 0.28% in the standard deviation of FHB severity and an indirect increase 0.31% via anther retention. This demonstrates firstly that the moderate correlation detected between plant height and FHB severity was mainly due to anther retention, and secondly that plant height per se has a high impact on FHB severity, even when the experiments are inoculated from above like in this study. As implication, plant height should therefore be considered simultaneously with anther retention in a multiple-trait selection approach for the development of semi-dwarf FHB-resistant cultivars.

### Combined ST-GWAS and MT-GWAS revealed two major *Rht* genes and novel pleiotropic loci for plant height, FHB severity and anther retention

Frequency distributions (Fig. 2) already indicated that all traits analysed here were quantitatively inherited, *i.e.* by many loci of varying effects. Indeed, a total of eight loci that control plant height were identified, with corresponding genomic regions on chromosomes 2A, 3A, 3B, 4D, 5B and 6A. Genomic regions of 4D and 6A were attributed to the presence of *Rht-D1b* and *Rht24b* (Würschum et al. 2015, 2017; Herter et al. 2018; Khadka et al. 2021; Tian et al. 2022), respectively, explaining 45.31% and 10.81% of the total genetic variation of plant height. This supports findings of Würschum et al. (2017), Herter et al. (2018) and Tian et al. (2017), who found that *Rht24* was the second most important *Rht* gene in Central European and worldwide commercial wheat breeding programmes. *Rht-D1* and *Rht24* are, respectively, gibberellin-insensitive (De Velde et al. 2021) and gibberellin-sensitive (Tian et al. 2022) genes responsible for reduced plant height in wheat. The other genomic regions were also enriched with several medium- and small-effect QTL which contribute to plant height. Those small-effect QTL represent the genomic background which explains existing genetic variation of plant height within *Rht* groups.

In addition to *Rht-D1*, a larger number of small-loci controlling FHB severity were identified on other genomic regions such as 2A, 2B, 4A, 5A, 6A, 6B and 7B, which were considered as genomic background contributing the variation of FHB severity among semi-dwarf genotypes. Several FHB resistance QTL were also reported on chromosomes 2A (He et al. 2016a; Gadaleta et al. 2019), 2B (Sari et al. 2018; Ollier et al. 2020), 4A (He et al. 2016a; Zhang et al. 2021; Nannuru et al. 2022), 5A (Steiner et al. 2019a; Ruan et al. 2020; Sari et al. 2020; Nannuru et al. 2022), 6A (Ruan et al. 2020), 6B (Cuthbert et al. 2007; Ollier et al. 2020) and 7B (Sari et al. 2018; Ollier et al. 2020). In addition, FHB resistance QTL on chromosomes 5A and 7B explained more than 7% of the genetic variation each and can be considered as important locally adapted QTL to improve FHB resistance in European winter wheat. The QTL on chromosome 5A was linked to marker (*AX-158558712*) at position 521 Mbp (Table S6). This falls within the QTL region *Qfhb.nmbu.5A.1* (480–552 Mbp) which was reported through a meta-QTL analysis by Venske et al. (2019) and recently detected in the European wheat panel by Nannuru et al. (2022). For anther retention, in addition to *Rht-D1b*, several medium- and small-effect QTL were detected in genomic regions including chromosomes 2A, 3A, 4A, 5A and 7A. Previous studies also identified QTL for anther retention on chromosomes 3A (Muqaddasi et al. 2019), 4A (Buerstmayr and Buerstmayr 2015) and 5A (Buerstmayr and Buerstmayr 2015; Steiner et al. 2019a; Sari et al. 2020; Xu et al. 2020). These QTL, representing the genomic background, contribute to the adjustments of anther retention, particularly within *Rht* genes groups.

In contrary to *Rht24b*, *Rht-D1b* exerted an opposite or interaction effect on both plant height and FHB severity. *Rht-D1b* explained more than 26% of the observed negative genotypic correlation between the two traits. The linkage disequilibrium analysis revealed an absence of close association between QTL identified for the different traits. This exhibits *Rht-D1* as a major gene conveying a negatively pleiotropic effect on FHB resistance. As indicated by Raherison et al. (2020), alleles of negatively pleiotropic loci exert favourable effects on one trait and unfavourable effects on the other trait depending on the breeding objectives. In commercial wheat breeding, the reduction effect of *Rht-D1b* on plant height represents a favourable effect, while its increase effect on FHB severity or susceptibility is perceived as an unfavourable effect. Several studies also found that *Rht-D1b* was highly associated with FHB susceptibility (Srinivasachary et al. 2008; Mao et al. 2010; Lu et al. 2013; Buerstmayr and Buerstmayr 2016; He et al. 2016b; Prat et al. 2017; Hales et al. 2020; Zhang et al. 2020). Two other interaction effect loci were also identified in other regions of chromosomes 4D and 5A between plant height and FHB severity. The second causal locus on chromosome 4D with linked marker *BobWhite_rep_c48828_217* at position 195 Mbp was associated with FHB severity only, demonstrating the existence of mediated negatively pleiotropic loci that contribute to the negative correlation between FHB resistance and plant height. Mediated pleiotropy as described by Hackinger and Zeggini (2017) represents a situation where a causal locus controls one trait, which in turn causes a second trait, resulting in significant association between the two traits as detected by correlation/covariance analysis. Moreover, our results also revealed that the small-effect loci identified for FHB severity on chromosomes 2A and 7B by the ST-GWAS and another locus on chromosome 6B conveyed a common effect on both FHB resistance and plant height. Although associated with FHB severity only, these QTL exhibited positive contribution to the interactions between the traits with about 16% of the genotypic correlation explained. Ghimire et al. (2022) also reported five stable and pleiotropic QTL associated with FHB resistance and related traits such as DON accumulation and plant height in red winter wheat. Similarly, Schulthess et al. (2017) reported the existence of genomic regions inducing pleiotropic effects on grain yield and related traits in wheat. Our study revealed the existence of positively pleiotropic loci which changes (increase or decrease) FHB severity and plant height in the same direction. These positively pleiotropic QTL can be exploited in breeding programmes to reduce FHB severity (i.e. improve FHB resistance) and plant height simultaneously. Most importantly, the QTL on chromosome 7B linked to marker *AX-158601365* at position 661 Mbp, explaining more than 7% of the genetic variation of FHB severity and exerting a positively pleiotropic effect on both FHB severity and anther retention represents a novel promising QTL which could be integrated into multiple-trait breeding strategy for higher FHB resistance in European winter wheat. Breeder-friendly KASP markers can be developed to facilitate the integration of this QTL into marker-assisted selection.

The high positive correlation and direct path effect observed between FHB severity and anther retention were controlled by the pleiotropic loci identified between FHB severity and plant height, particularly *Rht-D1* and the three QTL distributed on chromosomes 4D, 2A and 7B. All pleiotropic loci exerted common effects on FHB resistance and anther retention with about 24% of genotypic correlation explained in total. This firstly supports the existence of shared small-effect QTL between FHB resistance and anther retention (Lu et al. 2013) and secondly demonstrates that shared QTL may have positively pleiotropic effects on the two traits. Moreover, this positive pleiotropy can be either direct or mediated as the causal pleiotropic locus on chromosome 7B was specific to FHB severity, while others were associated with both traits. Existence of positive pleiotropy between FHB severity and anther retention offers the opportunity of utilizing genomics-assisted breeding to improve both FHB resistance and anther retention in winter wheat. Considering that the identified pleiotropic loci were small-effect QTL, except *Rht-D1*, the efficient exploitation of positive pleiotropy for improving FHB severity and anther retention could be achieved by implementing multi-trait genomic prediction as reported by Gaire et al. (2022) for FHB-related deoxynivalenol accumulation in wheat.

Furthermore, the moderate negative correlation observed between plant height and anther retention was controlled by *Rht-D1* and the two pleiotropic loci on 2A and 5A. *Rht-D1* and QTL on 5A exhibited negatively pleiotropic effects on plant height and anther retention, while the QTL on 2A exerted a mediated positively pleiotropic effect on the two traits. *Rht-D1* explained more than 56% of observed correlation between these traits, indicating that *Rht* genes had a major impact on anther retention and supporting that *Rht-D1b* was associated with high anther retention as recently reported by Buerstmayr and Buerstmayr (2022).

### *Rht24b* has no effect on FHB severity and anther retention

The combination of *Rht-D1b* and *Rht24b* was more frequent in our cultivars as previously reported within European wheat germplasm (Würschum et al. 2017; Tian et al. 2019). The similar FHB severity observed between *Rht24b* and genotypes with tall alleles (NoRht) is an indication that *Rht24b* did not affect FHB resistance (Herter et al. 2018; Miedaner et al. 2022). Similarly, our results also revealed that *Rht24b* did not significantly contribute to anther retention. Accordingly, no marker was found that co-segregates with the other morphological traits (Fig. 3). This offers the possibility of developing semi-dwarf genotypes with improved FHB resistance and low anther retention. However, the effect of *Rht24b* allele (− 3.42 cm) on plant height was about twofold lower than the effect of *Rht-D1b* (− 7.85 cm, Fig. 7a), showing that *Rht24b* exerted a lower reduction effect on plant height compared with other *Rht* genes (Tian et al. 2017; Herter et al. 2018). In addition, the high genetic variation for FHB severity observed among genotypes with *Rht24b* indicates the random existence of FHB-susceptible genotypes within this group. With this, alternative sources of resistance including locally adapted loci could also be explored to develop FHB-resistant genotypes within each *Rht* gene group, depending on breeding objectives.

### Genomic background has the potential to improve FHB resistance in genotypes with *Rht-D1b*

Considerable genetic variation was observed for FHB severity and anther retention among genotypes with *Rht-D1b* and *Rht24b* + *Rht-D1b* alleles, demonstrating that there is room for the development of FHB-resistant cultivars with the *Rht-D1b* allele. This large genetic variation in semi-dwarf genotypes can be attributed to the effects of genomic background distributed across several genomic regions. A key implication is that the genomic background has the ability to counterbalance the negative effect of *Rht-D1b* on FHB resistance and anther retention as explained by Buerstmayr and Buerstmayr (2022) who demonstrated by backcrosses with four near-isogenic lines (NILs) that the background resistance of the lines reduced efficiently the effect of semi-dwarfing alleles on FHB severity in spring wheat. Brar et al. (2019) also found that the genomic background and epistatic interactions had a significant impact on the expression of FHB resistance in hard red spring wheat. The more negative the genomic background effect, referring to higher resistance genomic background, the lower the FHB severity and anther retention. This offers a great opportunity to exploit the genomic background for improving FHB resistance in dwarf genotypes.

Effects of the genomic background on FHB severity and anther retention can be efficiently evaluated by calculating the genomic estimated breeding values (GEBV) of genotypes based on the effects of all loci, except *Rht* genes in our case (Bonnett et al. 2022). Several studies have suggested the stacking of additive effects or favourable alleles of QTL from genome-wide association study as an efficient approach to improve FHB resistance and related traits (Miedaner et al. 2006; Sidhu et al. 2020; Ghimire et al. 2022; Nannuru et al. 2022). However, our results showed that despite the existence of moderate to high correlations (*r* > 0.6) between the GEBV-based and stacking of additive effects (SAE) approaches, many genotypes with similar additive effects from GWAS exhibited different GEBV and FHB severity. This shows the limitations of SAE to accurately discriminate among genotypes based on their genomic background contrary to the GEBV-based approach and hence confirms the superiority and efficiency of the genomic prediction over fixed effects selection methods as reported by several previous studies (Juliana et al. 2017, 2022; Sandhu et al. 2021). In addition, the higher accuracy observed for the genomic prediction compared with marker-assisted selection based on the effects of QTL with p_G_ ≥ 5% from the ST-GWAS (Fig. 5) is another indication that genomic prediction would help to better exploit genomic background than GWAS. This poor performance of GWAS can be explained by the quantitative nature of FHB resistance and anther retention (Liu et al. 2019; Ruan et al. 2020), and that genomic background is constituted of several medium- and small-effect QTL. However, the genome-wide association study (GWAS) can identify only a small number of those minor QTL, not regarding the remaining which are also part of the genomic background and could have together a significant contribution to the phenotype. Moreover, using the GEBV-based approach, the best two genotypes with *Rht-D1b* had a FHB severity of 20.3 and 20.8%, respectively (Table 6). The best two genotypes without dwarfing alleles (Carimulti, Helmond, Table S1) had a FHB severity of 12.4 and 12.8%, respectively. Hence, *Rht-D1b* still has a penalty for FHB resistance of about 40% of increase in disease severity compared to genotypes without this semi-dwarfing allele. This illustrates the necessity to use alternative *Rht* alleles including *Rht24b*, boosted by a short genomic background or other FHB-neutral *Rht* dwarfing alleles.

Furthermore, the GEBV approach would select Anapolis as one of the best genotypes which exhibited the highest anther retention (99.6%). This is a typical cleistogamous genotype which possesses a structural floral barrier for pathogens, resulting in low FHB severity (Kubo et al. 2010; Tang et al. 2020; Buerstmayr et al. 2021; Zajączkowska et al. 2021). Similar observations were made in barley by Kawada and Kubo (2008) who found that cleistogamous genotypes had high FHB resistance and low mycotoxin accumulation.

## Concluding remarks

A clear understanding of the complex interactions between FHB severity and morphological traits and their genetic architecture can significantly contribute to addressing the needs for semi-dwarf wheat genotypes with high FHB resistance. The existence of high direct and indirect path effects between FHB severity and morphological traits demonstrates that multiple indirect trait selection for FHB severity has a great potential and should always integrate anther retention and plant height as important secondary traits. Positively direct and/or mediated pleiotropic loci controlling complex interactions between traits could be integrated into breeding programmes for an efficient multiple-trait selection for higher FHB resistance in wheat. Genomic background (GB) explained a high proportion of the genetic variance of FHB severity and anther retention and has the potential to increase FHB resistance in genotypes with *Rht-D1b*. Depending on the breeding goals, the development of FHB-resistant cultivars should consider *Rht24b* which was not linked to FHB susceptibility and exploit the GB for higher FHB resistance to counterbalance the negative effect of *Rht-D1b*. Similarly, PH could be further reduced in the *Rht24* group by the exploitation of GB. Strategies to efficiently exploit existing interactions among traits and the GB in breeding programmes to develop FHB-resistant cultivars with *Rht-D1b* should include the selection of: (i) taller genotypes within the *Rht-D1b* group as long as they have a high (negative) FHB-related resistance GB (e.g. Faktor, Mikon, Table 6), (ii) genotypes having the lowest anther retention, exploiting the high direct path and positively pleiotropic loci between FHB severity and anther retention, and/or (iii) genotypes with anther retention higher than 95% indirectly taking advantage of the contribution of cleistogamy to passive FHB resistance (e.g. Anapolis). Such genotypes could be most efficiently detected by using genomic estimated breeding values for FHB severity.

## Supplementary Information

Below is the link to the electronic supplementary material.

Supplementary file1 (XLSX 52 KB)

Supplementary file2 (PDF 914 KB)

## Data Availability

All reference data that are not presented in this manuscript are available in the supplementary materials.
