# Supplementary material for: Separation of the effects of two reduced height (Rht) genes and genomic background to select for less Fusarium head blight of short-strawed winter wheat (Triticum aestivum L.) varieties
Source: Theor Appl Genet. 2022 Sep 24;135(12):4303–26. doi: 10.1007/s00122-022-04219-4 (PMC9734223; doi:10.1007/s00122-022-04219-4)
Supplement: Supplementary file 2 — Supplementary file2 (PDF 914 KB) [file 122_2022_4219_MOESM2_ESM.pdf]

Separation of the effects of two reduced height (*Rht*) genes and genomic background to select for less Fusarium head blight of short-strawed winter wheat (*Triticum aestivum* L.) varieties

Félicien Akohoue[1], Silvia Koch[1], Jörg Plieske[2], Thomas Miedaner[1*]

Supplementary Tables

Table S1: Best linear unbiased estimations (BLUEs) of the eight traits across five environments
See Excel file

Separation of the effects of two reduced height (*Rht*) genes and genomic background to select for less Fusarium head blight of short-strawed winter wheat (*Triticum aestivum* L.) varieties

Félicien Akohoue, Silvia Koch, Jörg Plieske, Thomas Miedaner*

Table S2: SNP markers associated with plant height, FHB severity and anther retention from single trait genome-wide association study (ST-GWAS) models

Model[a]	Marker	Chr	Pos (Mbp)	UA/FA	FAF	-LOG10(p)	p_G (%)	Additive effect	FDR(p)
**PH**									
1	*rs20873*	4D	19.19	G/T	0.49	68.79	45.21	–7.02	3.3E-65
1	*rs10110*	6A	398.61	A/G	0.61	14.26	10.81	–3.93	2.2E-06
1 & 2	*rs13233*	2A	628.21	T/C	0.94	9.51	5.54	5.72	1.6E-06
1 & 2	*rs11421*	3B	850.79	A/G	0.13	10.11	3.11	0.03	5.2E-07
1 & 2	*rs23089*	3A	63.46	A/C	0.66	11.13	2.74	–2.02	7.5E-08
1 & 2	*rs8777*	5B	6.42	C/T	0.13	8.25	1.83	–1.99	8.6E-03
2	*rs19377*	5B	21.97	G/A	0.31	8.13	0.40	1.52	3.2E-05
1 & 2	*rs13973*	5B	21.76	T/C	0.70	7.33	0.05	1.60	1.6E-04
Total							69.69		
**FHB severity**									
1	*rs20873*	4D	19.19	T/G	0.51	15.42	26.36	5.97	7.6E-12
1 & 2	*rs3647*	5A	521.42	G/A	0.60	9.43	7.07	4.15	1.1E-03
1 & 2	*rs5192*	7B	661.18	A/G	0.66	6.83	7.03	5.27	2.2E-03
1 & 2	*rs13165*	2B	19.47	A/G	0.94	9.00	5.00	1.76	1.0E-05
1 & 2	*rs7788*	2A	419.17	A/G	0.36	8.62	3.83	–1.13	1.2E-05
1	*rs11875*	6A	2.73	T/C	0.84	8.15	3.41	2.67	2.8E-04
2	*rs4867*	6B	695.72	A/G	0.58	7.85	2.42	3.52	7.1E-05
1	*rs17561*	2B	54.19	T/C	0.93	10.44	1.84	2.01	1.1E-03
2	*rs14988*	2B	65.86	G/A	0.11	7.47	1.66	–3.89	1.4E-04
1	*rs8786*	4A	748.35	A/G	0.18	8.00	1.16	–1.41	2.2E-03
1	*rs1410*	4A	11.72	G/A	0.51	8.75	1.02	–1.99	1.2E-05
Total							60.74		
**AR**									
1	*rs20873*	4D	19.19	T/G	0.51	8.17	20.96	12.00	3.9E-03
1 & 2	*rs3629*	4A	712.25	G/A	0.16	8.76	8.47	–8.22	1.2E-05
2	*rs8776*	3A	521.92	A/G	0.83	8.04	5.07	6.12	3.6E-05
1 & 2	*rs7788*	2A	419.17	A/G	0.36	10.43	3.21	–2.72	7.4E-07
2	*rs7921*	3A	700.86	A/T	0.20	10.20	3.12	–7.86	3.1E-07
1	*rs17724*	7A	694.67	A/G	0.16	9.48	2.21	–8.75	3.3E-06
1 & 2	*rs18356*	7A	207.68	G/A	0.54	8.40	0.71	1.02	2.0E-03
2	*rs10472*	5A	21.20	A/G	0.75	7.53	0.22	4.99	6.6E-04
2	*rs19553*	7A	654.74	C/T	0.42	7.32	0.20	0.71	1.6E-04
Total							44.17		

[a]: 1 = ST-GWAS$_1$ model where all markers were included, 2 = ST-GWAS$_2$ without markers linked to plant height on chromosomes 4D (*Rht-D1*) and 6A (*Rht24*); Chr = chromosome, Pos = physical position from the wheat reference genome RefSeq v.2.1, UA/FA = unfavourable allele /favourable allele, FAF = favourable allele frequency, -LOG10(p) = negative logarithm of p-value, p_G = proportion of genotypic variance explained, FDR (p) = false discovery rate p-value, PH = plant height, and AR = anther retention. Only -LOG10(p) and FDR (p) of ST-GWAS$_1$ were reported for significant markers which were common to both models.

Separation of the effects of two reduced height (*Rht*) genes and genomic background to select for less Fusarium head blight of short-strawed winter wheat (*Triticum aestivum* L.) varieties

Félicien Akohoue, Silvia Koch, Jörg Plieske, Thomas Miedaner*

Table S3: Genomic prediction ability (rMG) and accuracy (rMG/H) for the five cross-validation sets including all markers

Sets	PH (cm)		FHB severity (%)		AR (%)	
	rMG	rMG/H	rMG	rMG/H	rMG	rMG/H
1	0.78	0.79	0.66	0.69	0.63	0.65
2	0.76	0.77	0.67	0.70	0.76	0.78
3	0.87	0.88	0.77	0.80	0.71	0.73
4	0.74	0.75	0.67	0.70	0.69	0.71
5	0.71	0.72	0.73	0.76	0.70	0.72
Mean	0.77	0.78	0.70	0.73	0.70	0.72
SE	0.06	0.06	0.05	0.05	0.05	0.05

PH = plant height, FHB=Fusarium head blight, AR = anther retention, SE = standard error; average prediction ability and accuracy are highlighted in red. Prediction ability and accuracy were significant at $p<0.001$.

Table S4: Distribution of *Rht* genotypes within cross-validation sets

Sets	NoRht	*Rht-D1b*	*Rht24b*	*Rht-D1b+Rht24b*	Set size
1	30	11	21	18	80
2	20	11	20	29	80
3	16	17	16	31	80
4	11	10	30	29	80
5	18	11	24	28	81
Total	95	60	111	135	401

Table S5: Genomic prediction ability (rMG) and accuracy (rMG/H) for the five cross-validation sets based on genomic background only

Sets	PH		FHB severity		AR	
	rMG	rMG/H	rMG	rMG/H	rMG	rMG/H
1	0.48	0.48	0.51	0.53	0.52	0.54
2	0.45	0.45	0.54	0.56	0.68	0.70
3	0.42	0.42	0.61	0.64	0.64	0.66
4	0.52	0.53	0.43	0.45	0.55	0.57
5	0.58	0.59	0.56	0.58	0.57	0.59
Mean	0.49	0.49	0.53	0.55	0.59	0.61
SE	0.06	0.07	0.07	0.07	0.07	0.07

SE = standard error; average prediction ability and accuracy are highlighted in red. Prediction ability and accuracy were significant at $p<0.001$.

Table S6: Significant markers sequences and physical positions of candidate genes

See Excel file

Separation of the effects of two reduced height (*Rht*) genes and genomic background to select for less Fusarium head blight of short-strawed winter wheat (*Triticum aestivum* L.) varieties

Félicien Akohoue, Silvia Koch, Jörg Plieske, Thomas Miedaner*

Supplementary Figures

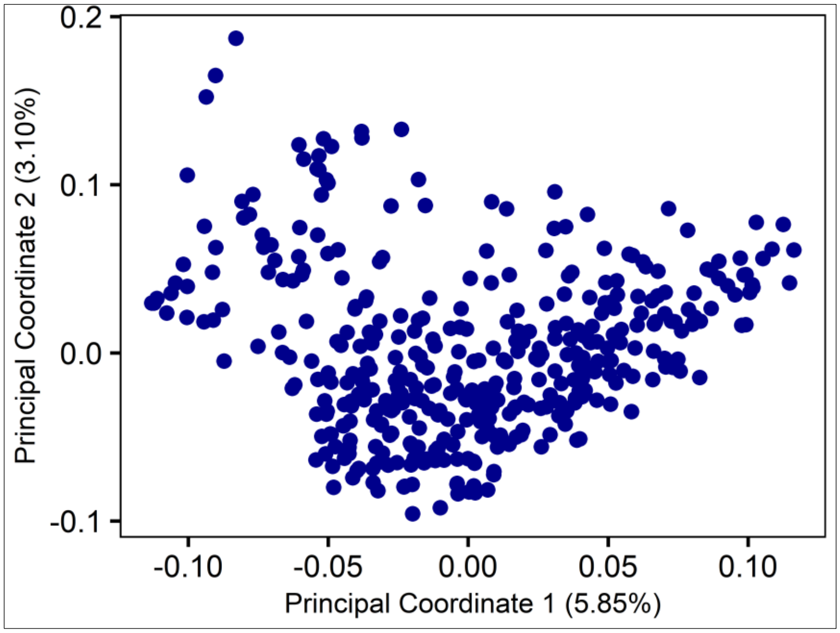

Fig. S1: Principal coordinate analysis of the 401 wheat genotypes included in the study

Separation of the effects of two reduced height (*Rht*) genes and genomic background to select for less Fusarium head blight of short-strawed winter wheat (*Triticum aestivum* L.) varieties

Félicien Akohoue, Silvia Koch, Jörg Plieske, Thomas Miedaner*

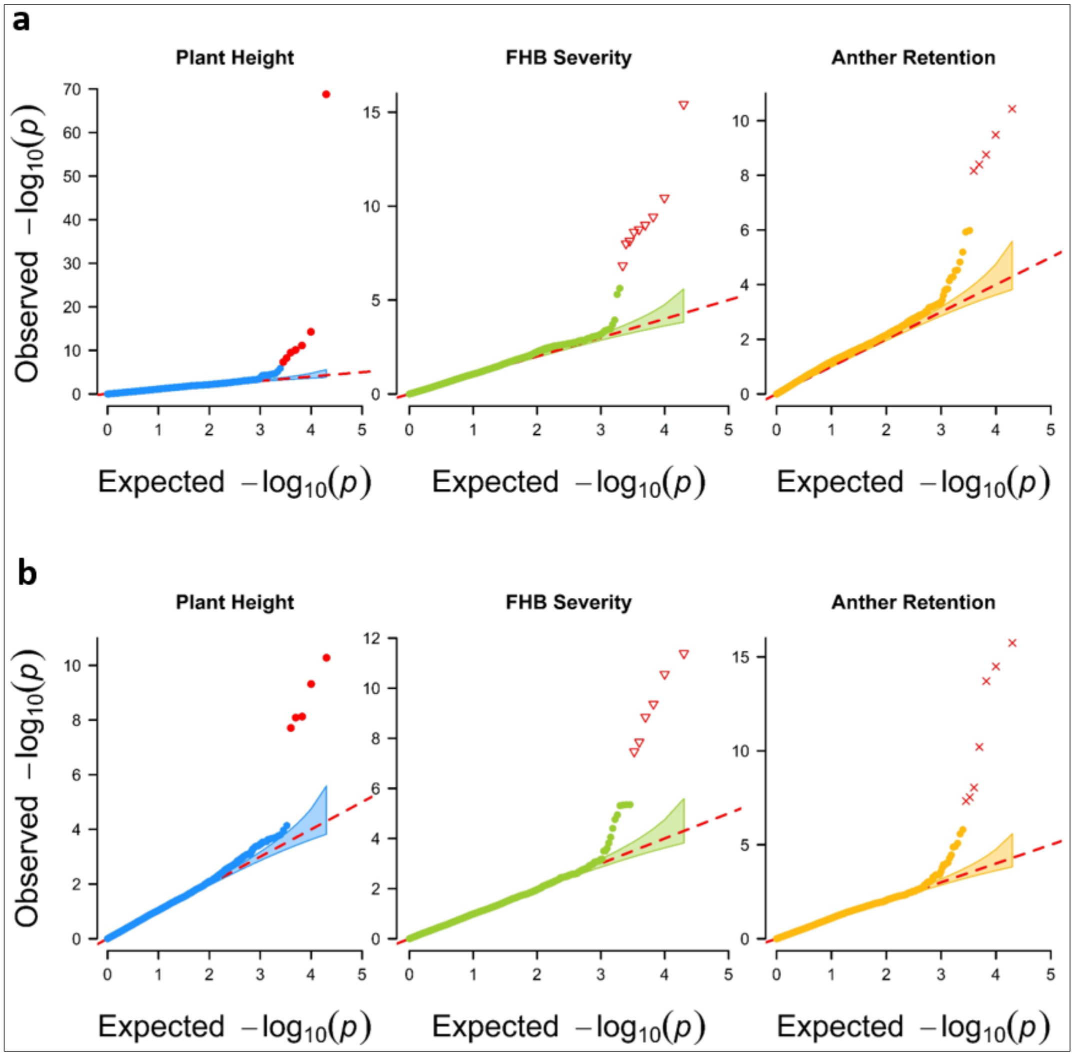

Fig. S2: Quantile-Quantile (Q-Q) plots showing the existence of true marker-trait associations (MTAs) from single trait genome-wide association studies (ST-GWAS): a = $ST\text{-}GWAS_1$ including all markers and b = $ST\text{-}GWAS_2$ without markers linked to plant height on chromosomes 4D (*Rht-D1*) and 6A (*Rht24*)

Separation of the effects of two reduced height (*Rht*) genes and genomic background to select for less Fusarium head blight of short-strawed winter wheat (*Triticum aestivum* L.) varieties

Félicien Akohoue, Silvia Koch, Jörg Plieske, Thomas Miedaner*

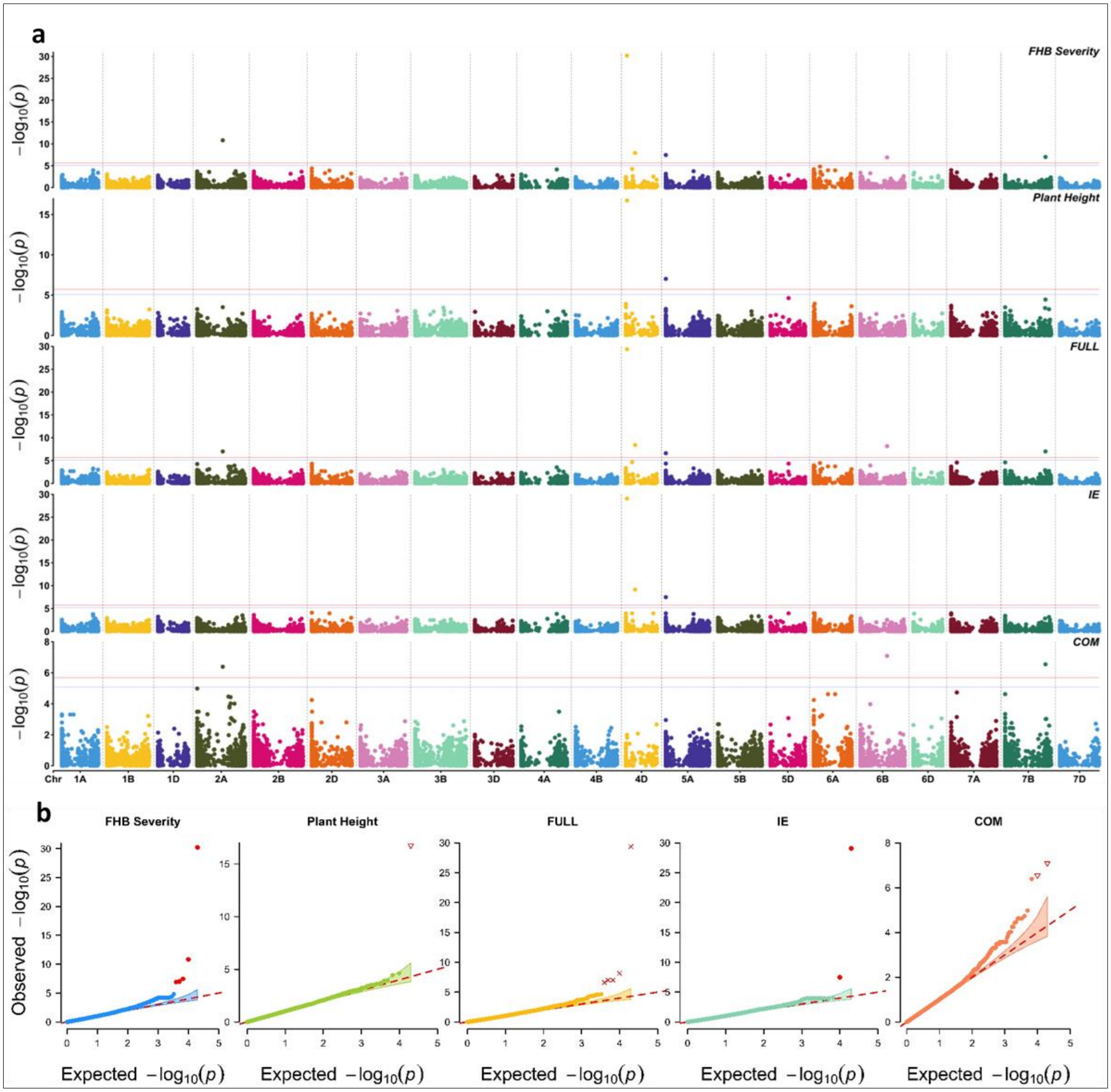

Fig. S3: Manhattan and Q-Q plots highlighting significant marker-trait associations (MTAs) for multi-trait genome-wide association studies (MT-GWAS): **a** = Manhattan plots of full model (FULL), common effects (COM), interaction effects (IE) between FHB severity and plant height; and **b** = Q-Q plots of full model (FULL), common effects (COM), interaction effects (IE) between FHB severity and plant height. The blue dotted line corresponds to an exploratory threshold of -LOG10(p) = 6 while the red plain line represents the Bonferroni corrected threshold cut-off of alpha = 0.01

Separation of the effects of two reduced height (*Rht*) genes and genomic background to select for less Fusarium head blight of short-strawed winter wheat (*Triticum aestivum* L.) varieties

Félicien Akohoue, Silvia Koch, Jörg Plieske, Thomas Miedaner*

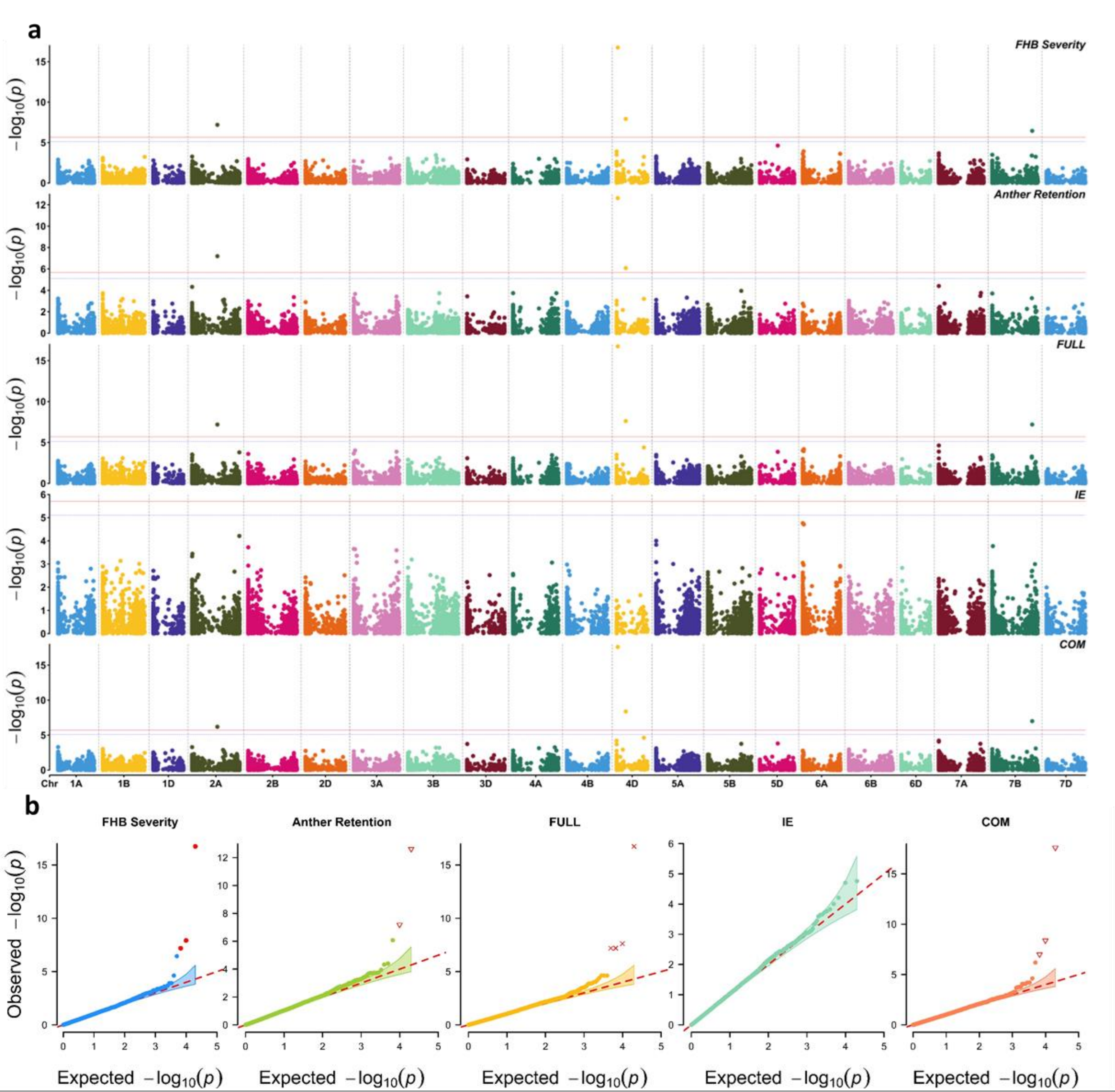

Fig. S4: Manhattan and Q-Q plots highlighting significant marker-trait associations (MTAs) for multi-trait genome-wide association studies (MT-GWAS). **a** = Manhattan plots of full model (FULL), common effects (COM), interaction effects (IE) between FHB severity and anther retention; and **b** = Q-Q plots of full model (FULL), common effects (COM), interaction effects (IE) between FHB severity and anther retention. The blue dotted line corresponds to an exploratory threshold of -LOG10(p) = 6 while the red plain line represents the Bonferroni-corrected threshold cut-off of alpha = 0.01

Separation of the effects of two reduced height (*Rht*) genes and genomic background to select for less Fusarium head blight of short-strawed winter wheat (*Triticum aestivum* L.) varieties

Félicien Akohoue, Silvia Koch, Jörg Plieske, Thomas Miedaner*

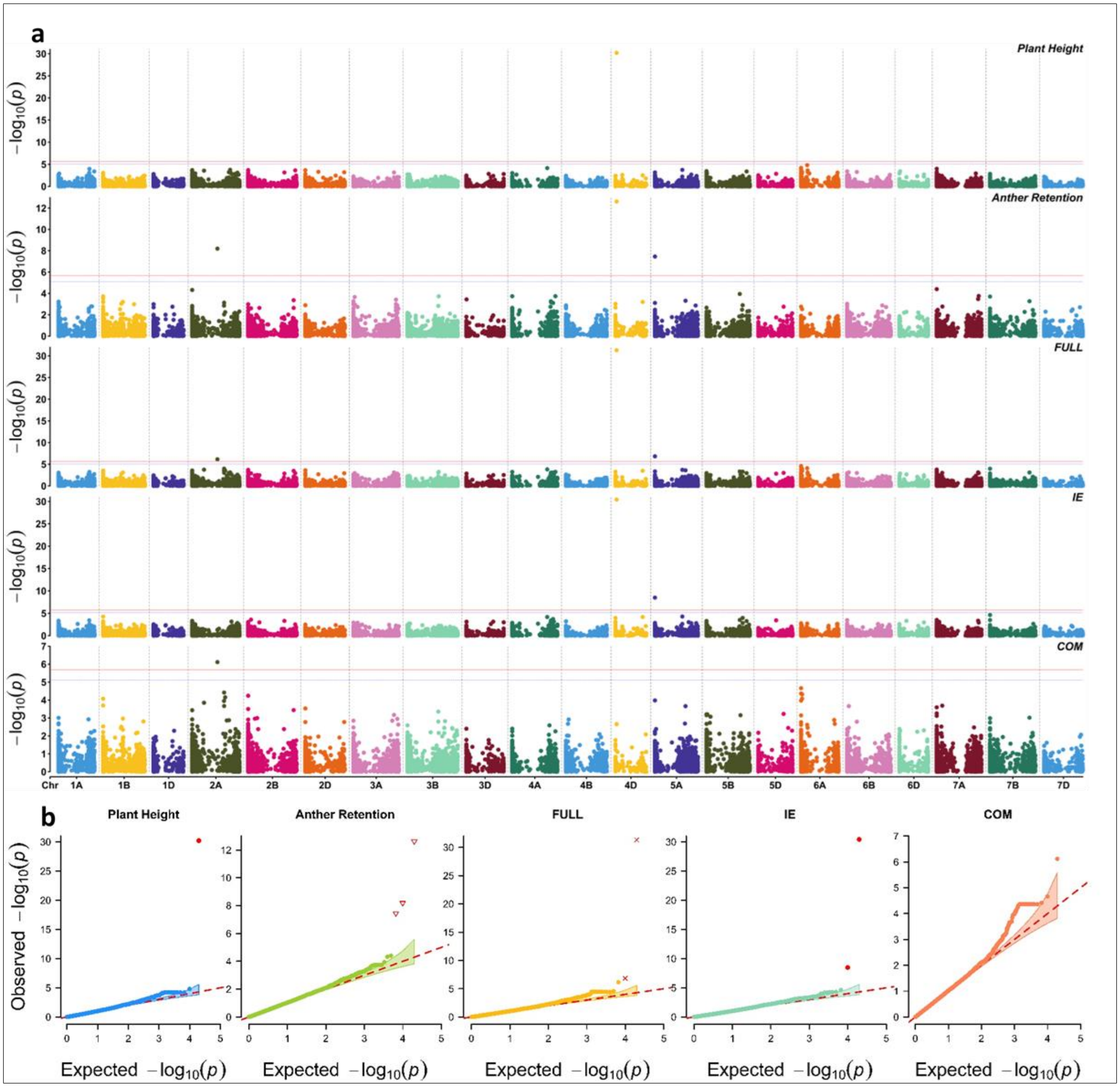

Fig. S5: Manhattan and Q-Q plots highlighting significant marker-trait associations (MTAs) for multi-trait genome-wide association studies (MT-GWAS). **a** = Manhattan plots of full model (FULL), common effects (COM), interaction effects (IE) between plant height and anther retention; and **b** = Q-Q plots of full model (FULL), common effects (COM), interaction effects (IE) between plant height and anther retention. The blue dotted line corresponds to an exploratory threshold of -LOG10(p) = 6 while the red plain line represents the Bonferroni-corrected threshold cut-off of alpha = 0.01

Separation of the effects of two reduced height (*Rht*) genes and genomic background to select for less Fusarium head blight of short-strawed winter wheat (*Triticum aestivum* L.) varieties

Félicien Akohoue, Silvia Koch, Jörg Plieske, Thomas Miedaner*

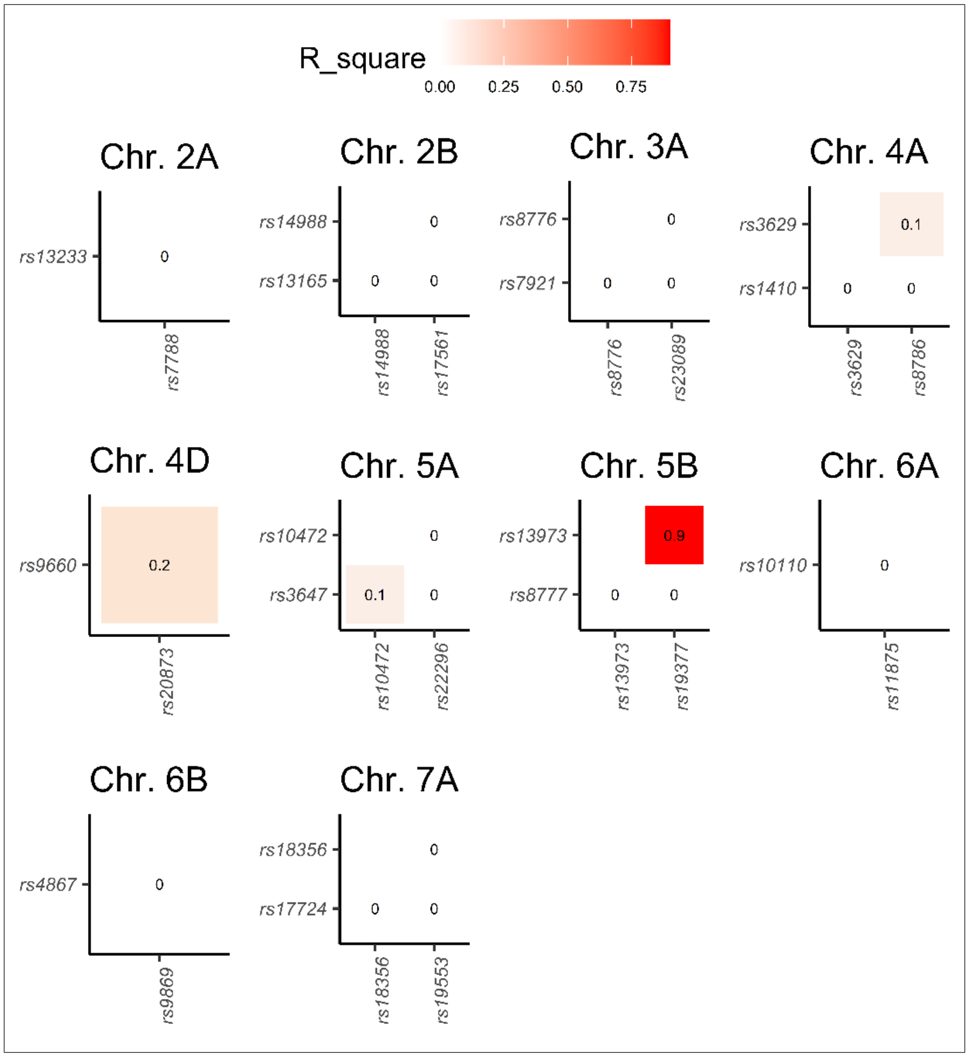

Fig. S6: Results of linkage disequilibrium among significant markers. Chr = chromosome
